# Formation of a Polycomb-Domain in the Absence of Strong Polycomb Response Elements

**DOI:** 10.1371/journal.pgen.1006200

**Published:** 2016-07-28

**Authors:** Sandip De, Apratim Mitra, Yuzhong Cheng, Karl Pfeifer, Judith A. Kassis

**Affiliations:** Program in Genomics of Differentiation, Eunice Kennedy Shriver National Institute of Child Health and Human Development, National Institutes of Health, Bethesda, Maryland, United States of America; CNRS, FRANCE

## Abstract

Polycomb group response elements (PREs) in Drosophila are DNA-elements that recruit Polycomb proteins (PcG) to chromatin and regulate gene expression. PREs are easily recognizable in the Drosophila genome as strong peaks of PcG-protein binding over discrete DNA fragments; many small but statistically significant PcG peaks are also observed in PcG domains. Surprisingly, *in vivo* deletion of the four characterized strong PREs from the PcG regulated *invected-engrailed* (*inv-en*) gene complex did not disrupt the formation of the H3K27me3 domain and did not affect *inv-en* expression in embryos or larvae suggesting the presence of redundant PcG recruitment mechanism. Further, the 3D-structure of the *inv-en* domain was only minimally altered by the deletion of the strong PREs. A reporter construct containing a 7.5kb *en* fragment that contains three weak peaks but no large PcG peaks forms an H3K27me3 domain and is PcG-regulated. Our data suggests a model for the recruitment of PcG-complexes to Drosophila genes via interactions with multiple, weak PREs spread throughout an H3K27me3 domain.

## Author Summary

Polycomb group proteins (PcGs) regulate growth and development of multi-cellular organisms by modifying histones and inhibiting chromatin remodeling resulting in gene silencing. PcGs act via binding to cis-regulatory DNA sequences known as Polycomb response elements (PREs). In genome wide studies, PREs are recognized as strong binding sites for PcG proteins, often near the promoter but many, weaker PcG binding sites are often spread throughout a PcG-domain. It was not known if these weak peaks were functional. We deleted the strong PcG peaks from the PcG-domain containing the *invected* and *engrailed* genes and show that a PcG-domain still forms. Further analysis showed these weak sites can act as PREs by recruiting PcGs and modifying histone H3. These weak peaks interact with each other and the target genes to maintain the three-dimensional structure of the PcG domain in the absence of strong peaks. We propose a model, where presence of several weak PREs along with strong ones ensures uniform distribution of histones modification over a PcG domain.

## Introduction

Polycomb group (PcG) proteins were first identified in *Drosophila* as repressors of homeotic genes [1], but genome-wide experiments over the last decade in *Drosophila* and mammals have identified hundreds perhaps thousands of other genes that are regulated by PcG proteins. PcG proteins work in complexes to modify chromatin [2, 3]. Two of the PcG complexes are PRC1 and PRC2. PRC1 contains the proteins Ph, Psc, Sce/Ring, and Pc and acts in part by inhibiting chromatin remodeling [4]. PRC2 contains Esc, Su(z)12, p55/CAF, and the histone methyltransferase E(z) that tri-methylates histone H3 on lysine 27 (H3K27me3). In *Drosophila*, PcG protein complexes are brought to the DNA by Polycomb group response elements (PREs) [2, 5]. In mammals, apart from three prominent cases, canonical PREs are largely absent [6–8] and in embryonic stem cells (ESCs), PcG complexes are recruited to clusters of unmethylated CpG-rich DNA sequences [9, 10]. In addition, a number of sequence-specific DNA binding proteins have been reported to recruit PcG proteins to specific genes in mammals [11–13].

PREs were discovered in transgenic assays in *Drosophila* and defined as DNA fragments that cause maintenance of silenced expression of a transgene [14–18]. Another assay for PRE activity is “pairing-sensitive silencing” (PSS) of the reporter gene mini-*white* in the *Drosophila* eye; repression of mini-*white* is much stronger when two copies of the PRE-reporter gene are present in the genome, either in cis or in trans [19, 20]. This ability of PRE-reporter transgenes to interact in order to increase silencing was the first indication of the ability of PREs to facilitate interactions between DNA fragments. PREs are now known to be involved in intra- and inter-chromosomal interactions, and to participate in setting up higher order chromatin structure [21–24], although insulators also play an important role in this [25].

Many different DNA binding proteins bind PREs and act together to recruit PcG protein, but how this occurs is poorly understood [26]. Pho was the first PRE DNA binding protein discovered and has been the most extensively studied [27]. In genome-wide chromatin-immunoprecipitation (ChIP) studies, PcG-targets are identified by the presence of H3K27me3-domains along with strong PcG-protein binding peaks at discrete sites within the target region [28]. These peaks of PcG-protein binding are known or presumed to be the PREs. Only a small fraction of PcG-binding peaks have been tested for PRE activity in transgenic assays, and only four PREs have been deleted or mutated in the *Drosophila* genome [29–31]. In all cases PRE deletion in situ led to unexpectedly weak phenotypes suggesting that other PREs or other mechanisms of PcG recruitment might be able to compensate for PRE loss but no conclusive study argue strongly against the popular yet poorly supported idea that in *Drosophila*, PcG target genes are regulated by a few strong PcG peaks.

In this study, we tested the functional importance of the well-characterized PREs of the PcG target genes *invected* (*inv*) and *engrailed* (*en*). *inv* and *en* are located next to each other in the genome, share regulatory DNA, and encompass a 113kb H3K27me3 domain [32]. Two PREs are upstream of *en* and were discovered by their ability to mediate PSS [19, 20]. Two other strong PcG peaks were identified upstream of *inv* based on ChIP-chip studies [33] and then later shown to have PRE activity in transgenic assays [34]. These 4 PREs are bound by PcG proteins and are the strongest PcG protein peaks in ChIP experiments in the *inv-en* domain in all cell types and developmental stages; hence, we hypothesized they would be absolutely required for PcG-repression in vivo. Contrary to this expectation, flies that had a deletion of all four strong *inv-en* PcG peaks maintain the wild type H3K27me3 domain, do not mis-express En in embryos or larvae, and survive to become fertile adults. These surprising results focused our attention on the many sites within the region that bind PcG proteins but at relatively low levels. We therefore performed several experiments to see if these were functional PREs. Chromosome conformation capture-sequencing (4C-seq) experiments in the *inv-en* domain suggest that these sites interact with each other and the *en* transcription unit. Further, a transgene that contains three weak PcG-protein binding sites, but no known PREs, establishes an H3K27me3 domain, recruits the PRC2 component E(z) to the transgene, is repressed in a pairing-sensitive manner, and is de-repressed in *pho* and *ph* mutants. Together our data suggest that *Drosophila* PcG-target genes are not just regulated by a small number of strong PREs, but rather, many DNA fragments within a target PcG domain can act as PREs. We suggest these PREs act together to recruit PcG proteins, and to spread the H3K27me3 mark.

## Results

### Characterized PREs from *inv*-*en* domain are not required for viability or formation of the H3K27me3 domain

**Fig 1:**
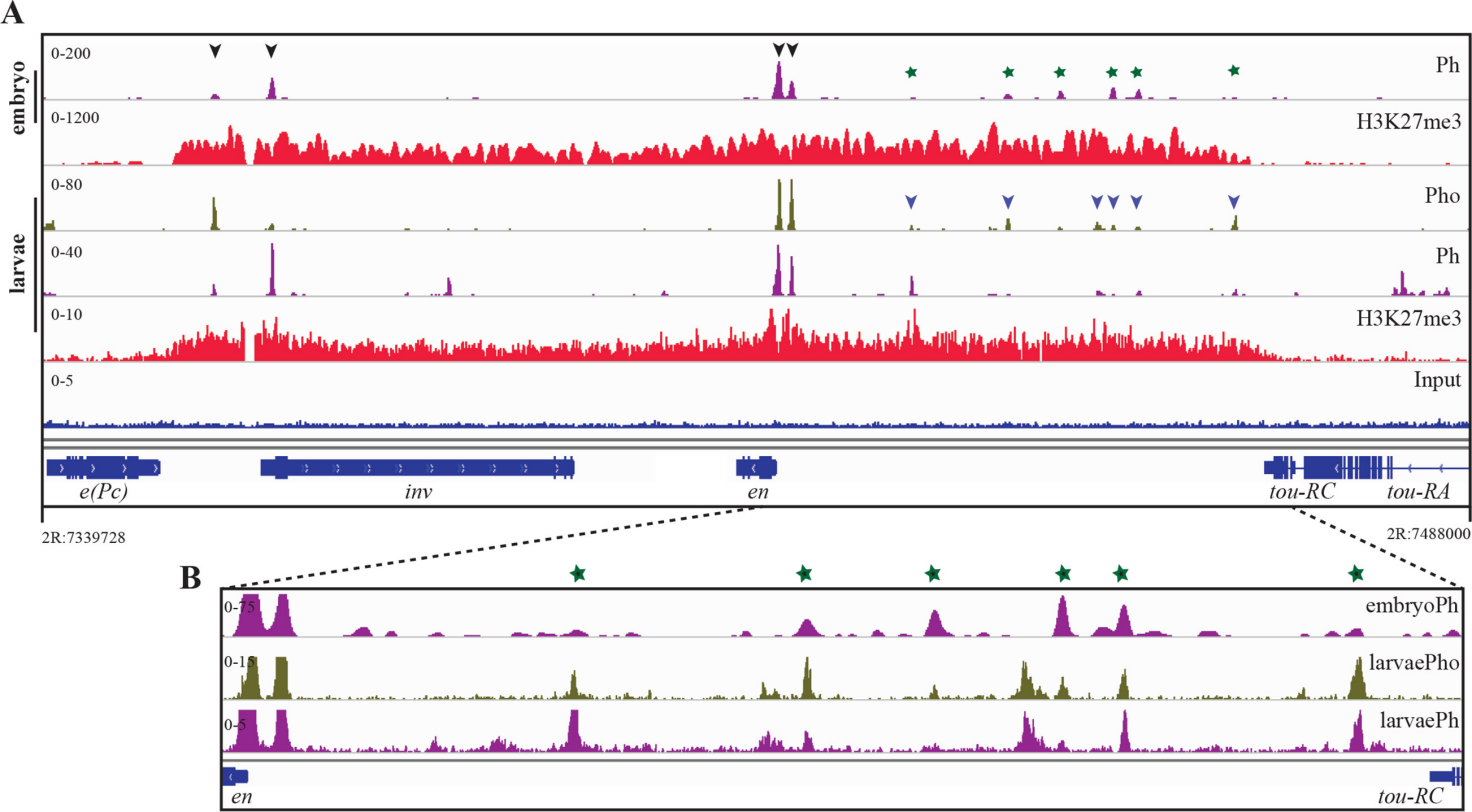
Pho, Ph and H3K27me3 ChIP-seq showing binding in the *inv-en* domain. (A) In embryo (top two rows) and larvae (middle three rows) the ChIP-seq distribution of Ph (Purple), Pho (olive green, larvae only) and H3K27me3 (red) over the *inv-en* domain are shown. Characterized PREs are indicated with black arrowheads; blue arrowheads highlight regions where Pho and Ph co-localize in larvae. Green stars highlight embryonic Ph binding sites that co-localize with larval Pho peaks. The genomic coordinates of the *inv-en* region are shown at the bottom (FlyBase release 5). (B) Zoomed in view upstream of the *en* transcription start site. The ChIP-seq scale has been changed to highlight the weak peaks (S1 Fig). Embryo ChIP-seq data was from Bowman et al. 2014 [38]. ChIP-chip experiments in embryos (Ph; [33]) and larvae (Pho; [41]) also showed the presence of smaller Ph and Pho peaks in the *inv-en* domain.

The *inv-en* genes are regulated by shared enhancers [32, 35] and comprise a ~113kb H3K27me3 domain (Fig 1). This domain extends from the 3’ end of the ubiquitously transcribed *enhancer of Polycomb* [*e(Pc*)] gene to the 3’ end of the ubiquitously transcribed *toutatis* (*tou*) gene. In S2 cells and adults there are four strong PcG-peaks in the *inv-en* domain [33, 36] and these correspond to the four PREs we have previously characterized [34, 37]. In order to identify the PREs within the *inv-en* region in larval tissues, we performed ChIP followed by next generation sequencing (ChIP-seq) with Ph and Pho antibodies (Fig 1). In agreement with previous studies on embryos and S2 cells [38], we find two large Ph and Pho peaks located just upstream of the *en* promoter (Fig 1). These two peaks are within DNA fragments that have PRE activity in reporter assays in embryos and pairing-sensitive silencing activity in the eye [19, 26, 37, 39]. In both embryos and larvae, there is a strong Ph peak at the *inv* promoter in addition to a weaker peak about 6kb further upstream. Pho is also present at these sites but the intensity of the Pho and Ph peaks varies at the sites, i.e. where the Pho peak is large, the Ph peak is small and vice versa (Fig 1). Similarly, DNA fragments that contain these two peaks have PRE activity in embryos and pairing-sensitive silencing of mini-*white* in the eye [34].

In addition to these strong peaks, there are a number of smaller, statistically significant peaks for both Ph and Pho, and many of these peaks overlap (Fig 1, blue arrow heads). To further investigate these binding sites of varying intensity, we analyzed the genome-wide Pho data and identified 3727 peaks at a p-value < 1e-5. Next, for the purposes of identifying bona fide Pho binding sites, these peaks were further refined using Peaksplitter [40] and the top ~20% (2324 peaks) were further analyzed (S1 Fig). We categorized these peaks into three subclasses according to their heights: the top 10% of the peaks (heights ~400) were defined as strong Pho peaks, the next 35% (100 ≤ peak heights ≤ 400) were termed weak Pho peaks, and even smaller peaks (heights < 50) were labeled as null Pho peaks (S1C Fig). The known PREs of the *inv-en* region coincided with 3 strong Pho peaks, and one in the second category, which is associated with a very strong Ph peak (Fig 1 and S1 Fig). In addition, there are 6 smaller, yet statistically significant Pho peaks that overlap with Ph peaks upstream of the *en* transcription start site, and they belong to the second category (S1 Fig). Interestingly, Ph ChIP-seq data from embryos [38] shows a similar pattern with strong peaks upstream of *en* and at the *inv* promoter, and several weak peaks, some that coincide with peaks observed in larvae (Fig 1A, green stars).

We wondered if the weaker peaks were dependent on the presence of the known PREs and if the weaker peaks were sufficient to render the *inv-en* domain PcG-regulated. To test this we deleted the known PREs from the endogenous *inv-en* domain hypothesizing that if these PREs were essential for PcG regulation of the locus, we would see disruption of the H3K27me3 domain and mis-expression (or loss of repression) of *en* and *inv* genes, most likely leading to severe developmental defects.

**Fig 2:**
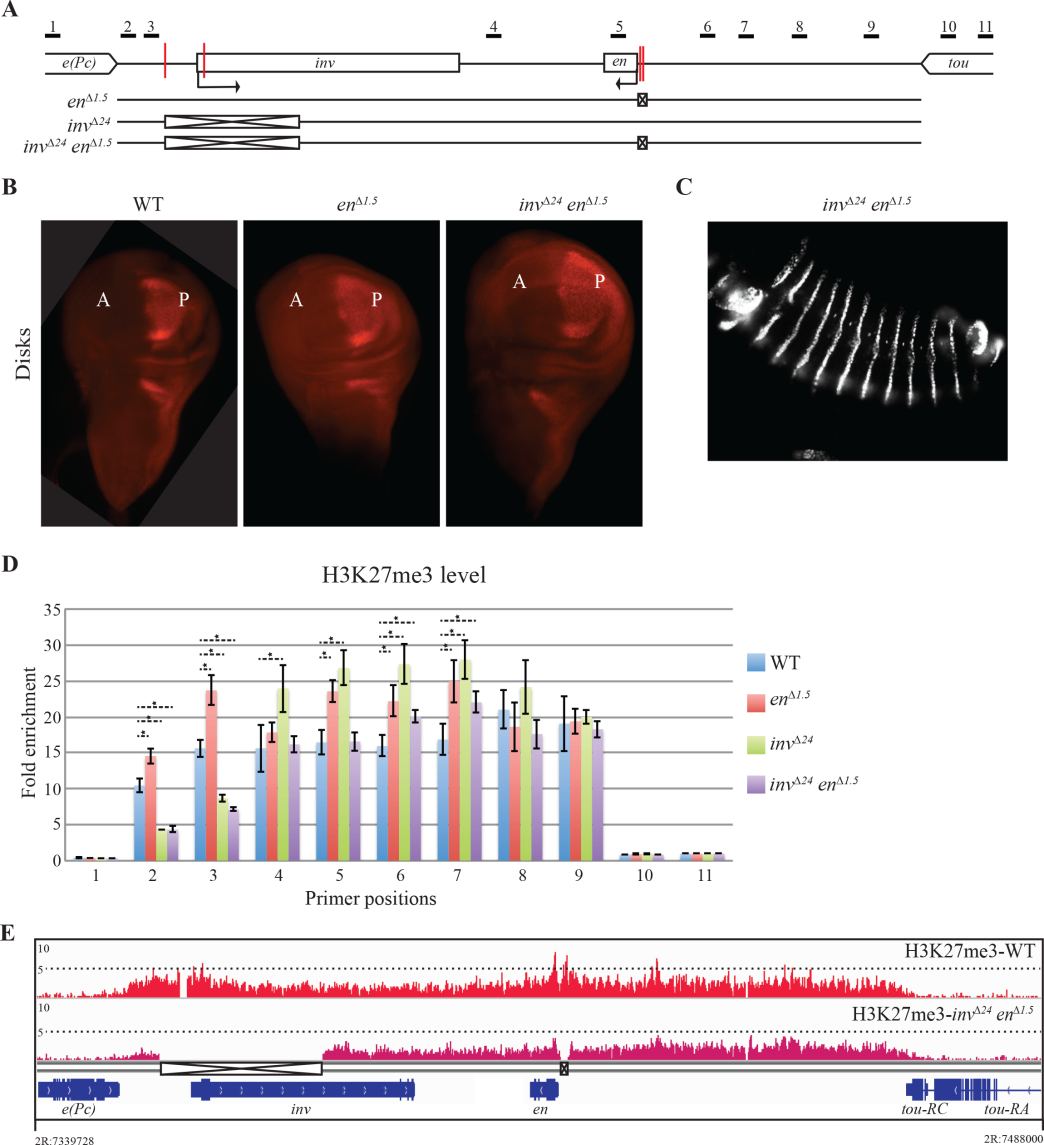
Removal of characterized PREs does not cause mis-expression of *en* or loss of the H3K27me3 domain. (A) Line diagram of the *inv-en* domain showing the extent of deletions in *en*^*∆1*.*5*^ (2R:7415804..7417265), *inv*^*∆24*^ (2R:7357207..7381178) and *inv*^*∆24*^*en*^*∆1*.*5*^ mutant flies using crossed boxes, characterized PREs are highlighted with red vertical lines. (B) Immunostaining with anti-En antibody in WT, *en*^*∆1*.*5*^ and *inv*^*∆24*^*en*^*∆1*.*5*^ imaginal wing disks. En is expressed in the Posterior compartment (P). PcG proteins repress En expression in the Anterior compartment (A). At least 30 individual wing discs were examined for each genotype. (C) Immunostaining with anti-En antibody in *inv*^*∆24*^*en*^*∆1*.*5*^ embryo. En is expressed in the stripes, note that there is no *en* mis-expression observed in between stripes. (D) Quantification of H3K27me3 by ChIP-qPCR over the *inv-en* domain in larval discs and brains of the genotypes listed. Small horizontal black lines in Fig 2A show regions amplified by qPCR. Results are shown as fold enrichment over background signal and are the average of three independent biological samples with three replicates each (mean±SEM). Statistical analysis of differential H3K27me3 accumulation was performed using Student’s t-test, P-values ≤ 0.05. Only the significant differences with WT are shown with ‘*’. (E) ChIP-seq distribution H3K27me3 in WT and *inv*^*∆24*^*en*^*∆1*.*5*^ over the *inv-en* domain is shown.

Three different mutants were used in our analysis. The first mutant, called *en*^*∆1*.*5*^, carries a 1.5kb deletion upstream of the *en* promoter that removes the two known *en* PREs ([42]; Fig 2A). These flies are homozygous viable and fertile indicating that repression of *inv-en* by PcG is largely normal. In fact, we directly examined expression in imaginal discs and did not detect any differences with wild type larvae (Fig 2B). The phenotypes we did observe in *en*^*∆1*.*5*^ flies were relatively minor. We initially observed a weak loss-of-function defect in wing veins in *en*^*∆1*.*5*^ flies [42], however that defect is no longer present in the stock and is now only revealed when *en*^*∆1*.*5*^ is put over a deficiency for the region (see below). Another phenotype, a defect in fusion of the cuticle at the midline in abdominal segments of the adult arose in multiple copies of this stock after about 3 years and is present in 45% of *en*^*∆1*.*5*^ flies (S2A Fig). Thus, the two PREs upstream of *en* are dispensable for viability, fertility, and there is no mis-expression of En in *en*^*∆1*.*5*^ imaginal discs.

The second mutation we analyzed, *inv*^*∆24*^, is a 24kb deletion that takes out both the characterized *inv* PREs and a large part of the *inv* transcription unit so that *inv*^*∆24*^ flies make no Inv protein. Since the *inv* gene acts redundantly with *en* and is dispensable in the laboratory, this fact did not interfere with our analyses. *inv*^*∆24*^ flies are viable and fertile and are phenotypically normal (S2A Fig), consistent with the idea that these two PREs are also not essential for PcG mediated repression of *en*.

Finally, we used P-element-mediated male recombination to generate a mutant with all four well-characterized PREs deleted (*inv*^*∆24*^*en*^*∆1*.*5*^). To our surprise, the double deletion flies are also homozygous viable and fertile, showing that the characterized PREs are dispensable in the laboratory (S2A Fig). Further, the expression pattern of En is normal in *inv*^*∆24*^*en*^*∆1*.*5*^ embryos and imaginal discs, suggesting that PcG-regulation of *en* is largely intact (Fig 2B and 2C).

We assayed the H3K27me3 levels over the *inv*-*en* domain by ChIP-qPCR in *en*^*∆1*.*5*^, *inv*^*∆24*^ and *inv*^*∆24*^*en*^*∆1*.*5*^ larval tissues and compared them to WT levels (Fig 2D). We used 11 primer sets that spanned the domain (Fig 2A). We also carried out ChIP-seq with anti-H3K27me3 antibody in wild type and in *inv*^*∆24*^*en*^*∆1*.*5*^ larvae (Fig 2E). In both analyses we saw that an H3K27me3 domain was established in mutant animals with H3K27me3 levels comparable to WT levels across almost the entire domain. We did see a modest reduction in H3K27me3 signal (about 50%) in the region just upstream of the *inv*^*∆24*^ deletion (see primer pairs 2 and 3 in Fig 2D). This may be a result of our mutagenesis protocol which leaves behind a PBac[WH] element (S2B Fig). PBac[WH] contains a Su(Hw) insulator element [43]; we suggest this insulator partially blocks the spreading of the H3K27me3 mark toward *e(Pc)* by PRC2. Altogether, these data show that deletion of the 4 characterized PREs from the *inv-en* domain does not disrupt the formation of the H3K27me3 *inv-en* domain, consistent with our data showing that En is accurately expressed in larval tissues.

### The size of the small Ph and Pho peaks does not change when the large peaks are deleted

**Fig 3:**
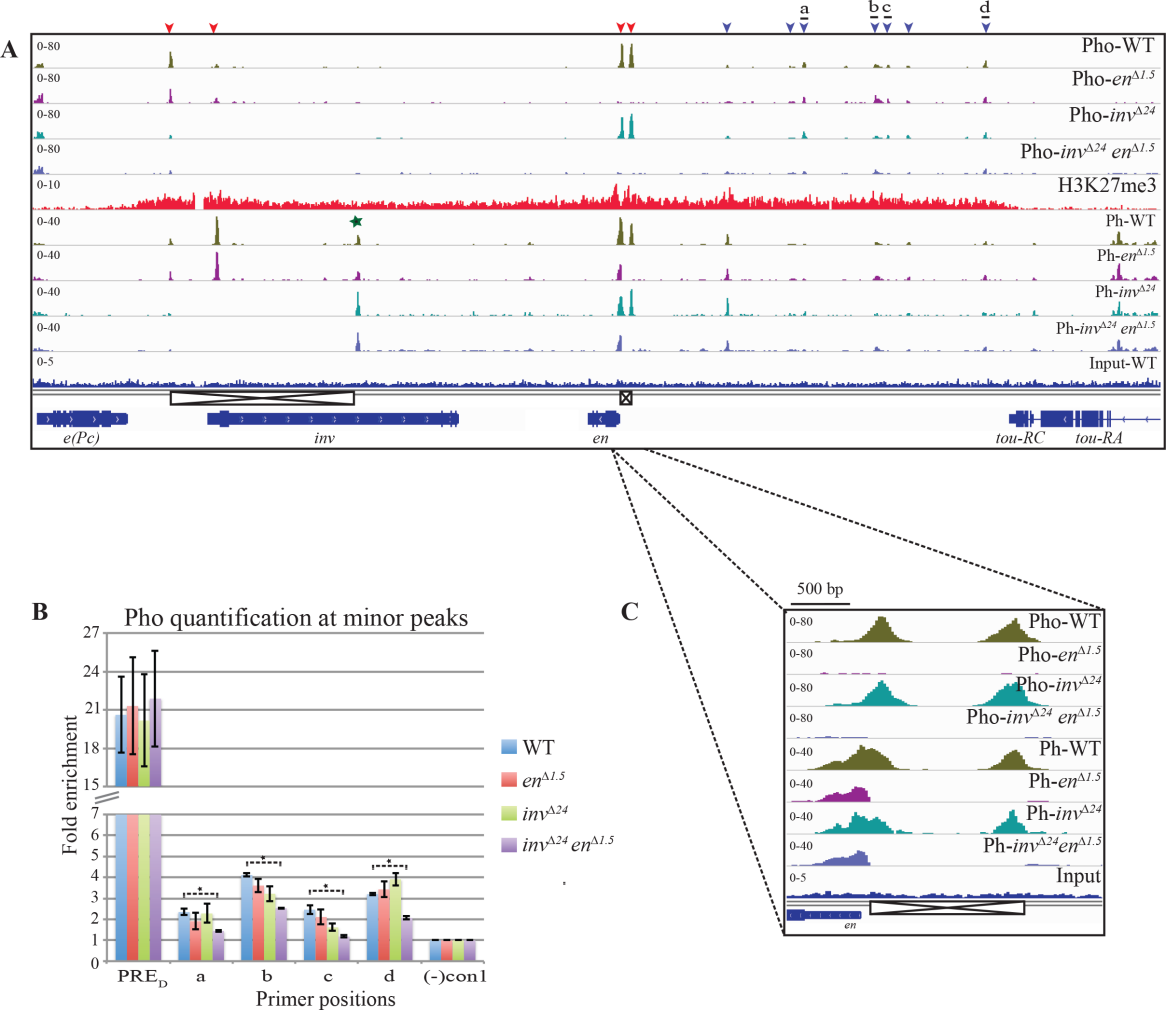
Deletion of the characterized PREs has little effect on Pho/Ph binding. (A) Upper four rows- ChIP-seq profile of Pho- in WT (olive green), *en*^*∆1*.*5*^ (purple), *inv*^*∆24*^ (cerulean) and *inv*^*∆24*^*en*^*∆1*.*5*^ (blue); middle row- distribution of H3K27me3 in WT (red); lower four rows- ChIP-seq profile of Ph- in WT (olive green), *en*^*∆1*.*5*^ (purple), *inv*^*∆24*^ (cerulean) and *inv*^*∆24*^*en*^*∆1*.*5*^ (blue) over *inv-en* domain. Positions of the genes (navy blue) are shown at the bottom. Deletions in the domain are shown using crossed boxes. (B) Quantification of Pho at 4 small peaks in WT, *en*^*∆1*.*5*^, *inv*^*∆24*^ and *inv*^*∆24*^*en*^*∆1*.*5*^. Small horizontal black lines in Fig A show regions amplified by qPCR. PRE_D_ (from *Ubx*) is used as positive control. Results are shown as fold enrichment over background signal and are the average of three independent biological samples with three replicates each (mean±SEM). Statistical analysis of differential Pho accumulation was performed using Student’s t-test, P-values ≤ 0.05. Only the significant differences with WT are shown with ‘*’. (C) A 2.5kb region including the *en* promoter and upstream region is enlarged.

We carried out ChIP-seq experiments with anti-Pho and anti-Ph antibody on WT, *en*^*∆1*.*5*^, *inv*^*∆24*^, and *inv*^*∆24*^*en*^*∆1*.*5*^ larvae. Strikingly, the smaller Pho and Ph peaks were still present after deletion of the large peaks (Fig 3A, blue arrowheads). ChIP-qPCR with Pho antibody confirmed these results and show that there is only a minimal reduction in Pho binding even in *inv*^*∆24*^*en*^*∆1*.*5*^ larvae (Fig 3B). These data show that the small peaks are independent binding sites, and do not result from cross-linking of these DNA fragments with the proteins bound to the large peaks. We therefore hypothesized that these small peaks might be true PREs and searched for the presence of consensus binding sites for PRE-DNA binding proteins and for the GAGA and GTGT motifs found in PREs within 1kb regions encompassing the peaks (S1 Table). All of the fragments contain consensus Pho sites and most contain consensus binding sites for a collection of PRE-DNA binding proteins, however we note that peaks 2, 3, 4, and 6 contain no GAGA sites, a core component of PREs, and thus are not canonical PREs [5].

Most Ph peaks co-localize with Pho peaks in the *inv-en* region, however there are some exceptions. There is a strong Ph- binding site near the middle of the *inv* gene where Pho is not bound; for an unknown reason, the intensity of this peak was increased in the *inv*^*∆24*^, and *inv*^*∆24*^*en*^*∆1*.*5*^ mutants (Fig 3A, green star). Examination of Pho and Ph binding upstream of En is interesting; the *en* promoter-proximal peak of Ph is much wider than that of Pho, and extends over the *en* promoter (Fig 3C). Promoter-proximal Pho binding is completely lost in *en*^*∆1*.*5*^ and *inv*^*∆24*^*en*^*∆1*.*5*^ larvae, but Ph was still present at the *en* promoter (Fig 3C) albeit at a lower level. We suggest that Ph is recruited to the *en* promoter via another DNA binding protein or via interaction with cohesin [44] or to paused RNA Polymerase bound to the *en* promoter.

### Phenotypes of *inv*^*∆24*^*en*^*∆1*.*5*^ flies

**Fig 4:**
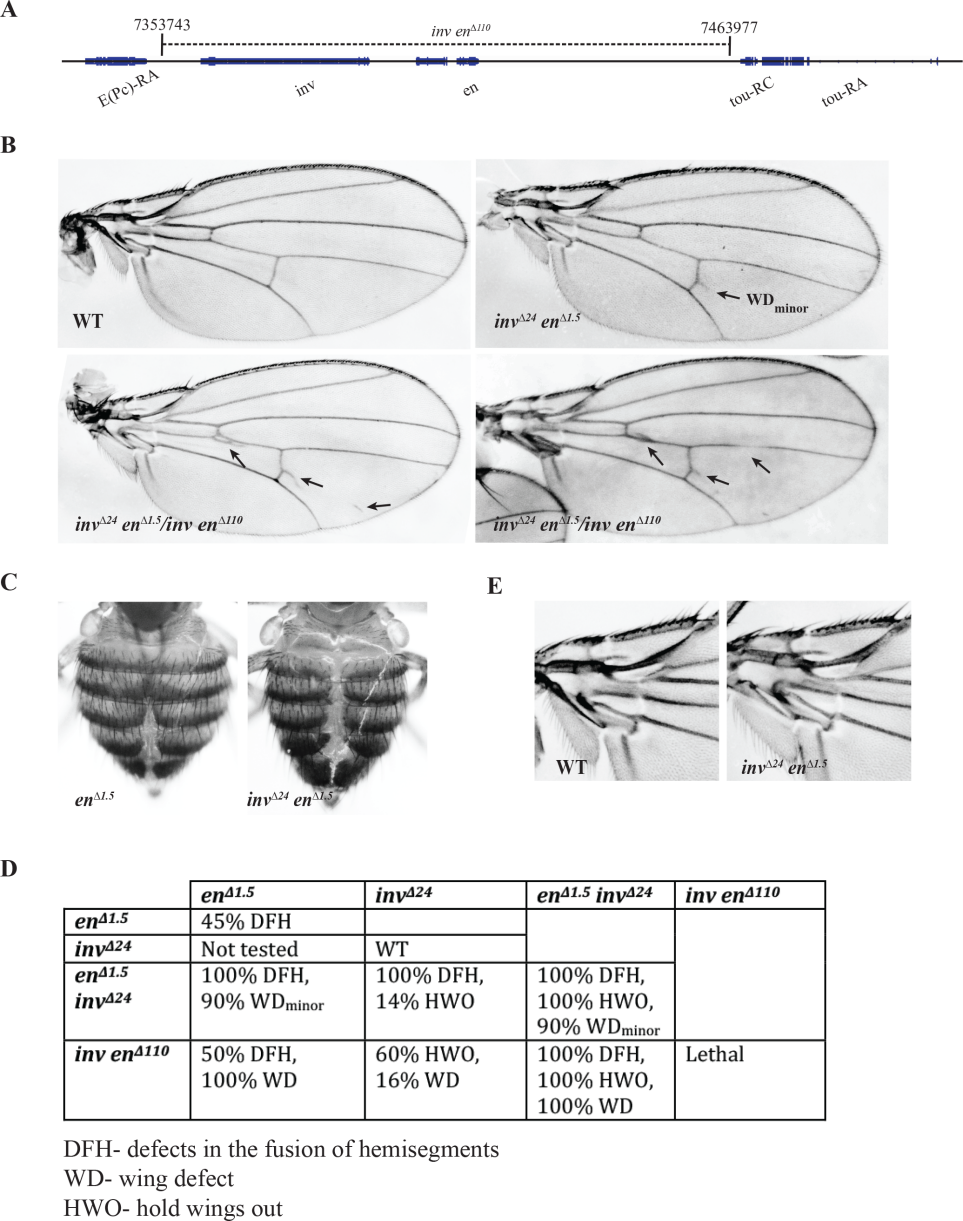
Phenotype categorization of *inv-en* deficiencies. (A) Schematic of 110kb deletion in the *inv-en* domain. (B) Pictures of adult wings of indicated genotypes. Defects in the wings are indicated with black arrows. (C) Zoomed-in view of adult female abdomen of *en*^*∆1*.*5*^ (left), *inv*^*∆24*^*en*^*∆1*.*5*^ (right) flies. (D) Categorization of different phenotypes of *inv-en* deficiencies. (E) Zoomed-in view of hinge region from WT and *inv*^*∆24*^*en*^*∆1*.*5*^ adult fly wings.

The fact that even *inv*^*∆24*^ *en*^*∆1*.*5*^ flies are viable and fertile indicates that PcG repression of *en* is fundamentally normal. However, Trithorax group protein complexes (TrxG, a transcriptional activator) also bind to PREs and it has therefore been proposed that PREs are also necessary for gene activation. We therefore examined more fully the phenotypes we did note in mutant flies to look for evidence of loss of *en* expression. Adult *inv*^*∆24*^*en*^*∆1*.*5*^ flies have three phenotypes: 1) they hold their wings out, 2) 90% of the flies have a very subtle defect in the posterior crossvein of the wing [WD_minor_, Fig 4B top right], and 3) there is a defect in the fusion of left and right dorsal hemisegments (Fig 4C). The first two phenotypes are recessive; and the third is dominant, and much stronger in the homozygote. En is required for anterioposterior patterning in the adult abdominal segments [45], but a role for En in hemisegment fusion has not been described; thus, the reason for this phenotype is unknown.

We generated a ~110kb deletion (*inv en*^*∆110*^) that almost entirely removes the *inv-en* region (Fig 4A) and crossed it to *en*^*∆1*.*5*^, *inv*^*∆24*^, and *inv*^*∆24*^ *en*^*∆1*.*5*^ flies. Our data strongly suggest that the wings held out phenotype is caused by a loss of *inv* function and the defective wing vein phenotype is caused by a loss of *en* function. *inv*^*∆24*^ flies have normal wings; this shows that *en* can fully substitute for *inv* in an otherwise WT fly. However, 60% of *inv*^*∆24*^*/inv en*^*∆110*^ flies hold their wings out, suggesting that loss of *inv* function strongly contributes to this phenotype (Fig 4D). In contrast, 100% of *en*^*∆1*.*5*^*/inv en*^*∆110*^ flies have wing defects in the posterior compartment, while none hold their wings out (Fig 4D). We examined the phenotype of the wing hinge in *inv*^*∆24*^ *en*^*∆1*.*5*^ mutants and could not see any abnormalities; thus it is likely that the wings held out phenotype is caused by a defect in muscles or in the innervation of the wings (Fig 4E).

The wing vein phenotypes we observe could be due to a decreased level of En expression. Therefore, we measured the level of *inv*, *en*, *tou*, and *e(Pc)* mRNA and quantified the En signal in wing imaginal discs of WT and *inv*^*∆24*^*en*^*∆1*.*5*^ and saw no significant differences (S2C–S2E Fig). The *inv* and *en* promoters are both associated with paused RNA Polymerase II (PolII), a characteristic of Polycomb-regulated genes [36, 46]. We tested whether deletion of the PREs had any effect on the level of PolII at the promoter of these genes [*inv* and *en*] and also on the flanking genes [*E(Pc)* and *tou*] in larvae. We found that a low amount of PolII is present at the promoters of these genes and this level did not change in the absence of characterized PREs (S2F Fig). Thus, while our genetic data suggests that *en*^*∆1*.*5*^ causes a decrease in *en* function, we could not detect any changes in the level or pattern of En expression in *inv*^*∆24*^ *en*^*∆1*.*5*^ wing discs. These data suggest that deletion of the characterized PREs causes only a subtle decrease the level of En expression. Since a promoter-tethering element, necessary for interaction with imaginal disc enhancers [47], is also present within the *en*^*∆1*.*5*^ deletion, we cannot attribute this subtle decrease to a loss of the PRE.

### Deletion of strong PREs does not affect local 3D structure of *inv-en* domain

As opposed to a hierarchical model of recruitment of Polycomb proteins to PREs [48], recent articles proposed a combinatorial recruitment of Pho at noncanonical, low-affinity Pho binding sites present within a Polycomb domain [49, 50]. Through their analysis of Hi-C data Schuettengruber et al. (2014) proposed that, preferentially within a Polycomb domain, different Pho sites contact each other forming a distinct compact structure [49]. To test whether the weak peaks interact with the *en* transcription unit, we carried out 4C-seq experiments in our WT and deletion mutants using a probe from the *en* transcription unit. We note that our 4C-experiment was done with larval brains and discs, and contains a mixture of cells with En ‘ON’ and those with En ‘OFF’. Thus, differences detected could indicate differences in either the ‘ON’ of ‘OFF’ state.

**Fig 5:**
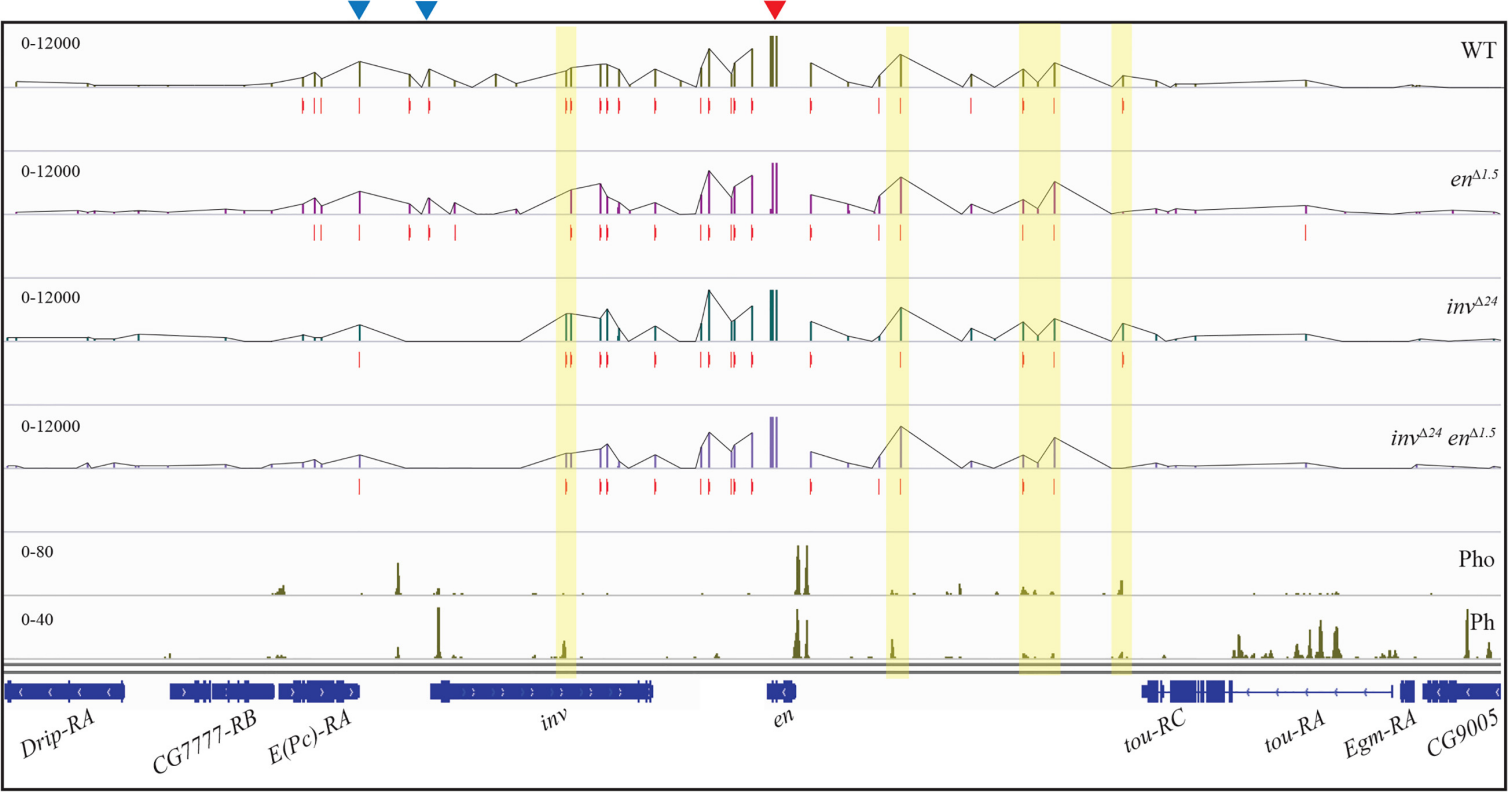
Deletion of strong PREs does not change the 3D structure of the *inv-en* domain. 4C interaction map at *inv-en* domain of WT, *en*^*∆1*.*5*^, *inv*^*∆24*^ and *inv*^*∆24*^*en*^*∆1*.*5*^, significant 4C interactions are indicated with red vertical lines. Distribution of Pho and Ph within the domain is shown in the last two rows. Viewpoint (bait) is indicated with red arrowhead on top. The interactions with the known *inv* PREs are indicated with blue arrowheads and the interactions with the weak Pho/Ph sites are highlighted with yellow transparent boxes. Positions of the genes (navy blue) are shown at the very bottom.

This bait is ~2.9kb downstream of the *en* promoter (Fig 5, red arrowhead). In concordance with previous reports [21, 44, 51], we observed an interaction of known *inv* PREs with the *en* transcription unit (Fig 5, indicated with blue arrowheads), the known *en* PREs are too close to the bait to analyze. In addition, we found interactions of the *en* transcription unit with several different fragments distributed within the *inv-en* domain with the strength of these interactions varying amongst the fragments; of these, some were interactions between weak Pho/Ph peaks with the *en* transcription unit (Fig 5, yellow shaded boxes). Some of the weak Pho/Ph peaks have higher levels of interaction with the *en* transcription unit than the known *inv* PREs. This suggests that these weak Pho/Ph peaks may play an important role in the establishment of the topology of this domain. We note that, in agreement with data on the *inv-en* domain in embryos [51], we did not observe any significant interaction with any other Polycomb domain.

### Establishment of an H3K27me3 domain over a transgene that contains weak Pho/Ph peaks

**Fig 6:**
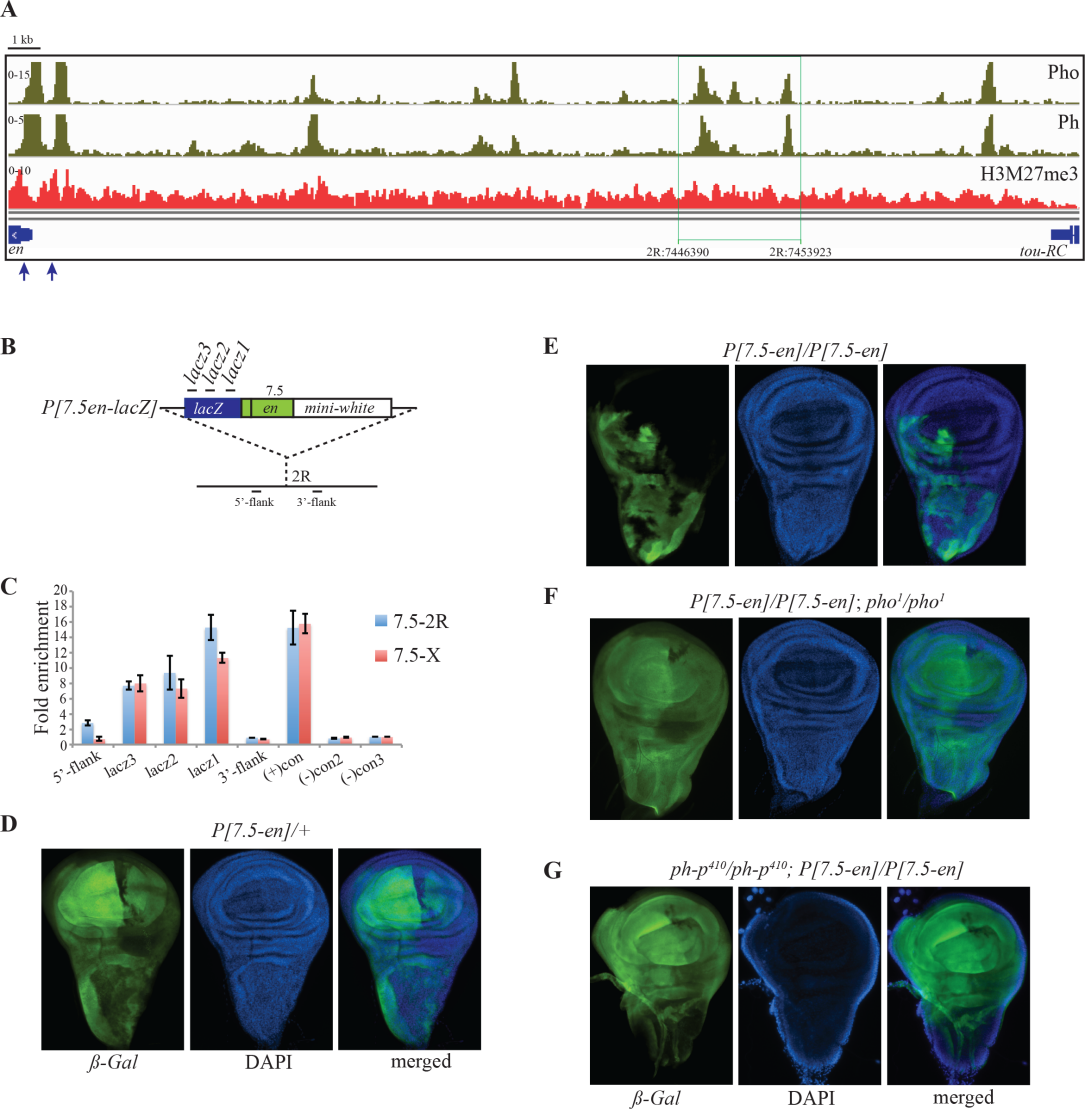
A transgene containing small Pho/Ph peaks is PcG regulated. (A) An overview of ChIP-seq distribution of Pho, Ph and H3K27me3. Characterized *en-*PREs are indicated with blue arrows. A 7.5kb fragment (2R:7446390..7453923) with weak Pho/Ph peaks used for transgenic assay is shown by green box. (B) Diagram of the transgenic construct containing the 7.5kb fragment. (C) Quantification of H3K27me3 over *lacZ* in two transgenic lines (7.5-2R and 7.5-X) is shown; primer sites are shown in B. H3K27me3 levels from fragments ~1kb upstream and downstream of the insertion site at chromosome 2R are also shown. Results are shown as fold enrichment over control signal and are the average of three independent biological samples with three replicates each (mean±SEM) (primer details in S2 Table). (D) ßgal and DAPI staining of wing imaginal disc from a larvae heterozygous for *P[7*.*5en-lacZ]*. (E) ßgal and DAPI staining of a wing imaginal disc from a larvae homozygous for *P[7*.*5en-lacZ]*. (F) ßgal and DAPI staining of a wing imaginal disc from a larvae homozygous for *P[7*.*5en-lacZ]* in a *pho*^*1*^ mutant. (G) ßgal and DAPI staining of a wing imaginal disc from a larvae homozygous for *P[7*.*5en-lacZ];* in a *ph-p*^*410*^ mutant.

We next examined whether a ~7.5kb fragment (2R:7446390..7453923) containing three weak Pho/Ph peaks (Fig 6A, region outlined with green line, Fragment R from Cheng et al., 2014 [32]) could cause accumulation of H3K27me3 over the *lacZ* gene when cloned into the P-element vector *P[en-lacZ]* (Fig 6B, [32]). This vector contains 400bp upstream of the *en* transcription start site and 139bp of the untranslated *en* leader, but no PREs. We examined the accumulation of H3K27me3 over the *lacZ* gene in larva with *P[7*.*5en-lacZ]* inserted at two different chromosomal insertion sites (2R:17047933, X:11744845). Our data show that the 7.5kb fragment is required to create a small H3K27me3 domain over the *lacZ* gene (see below); H3K27me3 is highest immediately adjacent to the *en* fragment and drops off rapidly within the *lacZ* gene (Fig 6C). The mark does not spread to regions flanking the transgene. We also carried out ChIP with anti-E(z) antibody in *P[7*.*5en-lacZ]-*2R larvae to determine whether the fragment with weak peaks could recruit this PRC2 subunit, our data show that there is an accumulation of E(z) at the junction of promoter-weak peak fragment and at the 5’ end of *lacZ* (S3A Fig).

We also examined the *ß-galactosidase* expression pattern in *P[7*.*5en-lacZ]* transgenic larvae from 2 independent insertion sites. *ß-galactosidase* was expressed in a variegated pattern in discs heterozygous for the insert (Fig 6D), and this variegation was extremely variable for the insert on the X. There was a dramatic increase in silencing in imaginal discs homozygous for *P[7*.*5en-lacZ]* inserted on the 2^nd^ chromosome (Fig 6E). The extreme variegation of discs from *P[7*.*5en-lacZ]-*X was also present in females homozygous for the transgene, and in this case, it was difficult to assess whether there was more silencing in homozygotes. Variegated expression and increased silencing of *lacZ* in wing discs homozygous for the transgene are both reminiscent of the effect of PREs on expression of the mini-*white* gene in the eye. In fact, *P[7*.*5en-lacZ]-*X homozygotes have a lighter eye color than heterozygotes, and thus show pairing-sensitive silencing. The eye color of *P[7*.*5en-lacZ]*-2 flies is variegated, but is darker in homozygotes. We tested the effect of *pho* and *ph* mutations on ß-gal expression in *P[7*.*5en-lacZ]*-2 wing imaginal discs. Silencing of *lacZ* from *P[7*.*5en-lacZ]*-2 is greatly reduced in a *pho* homozygous mutant (Fig 6F) suggesting that Pho plays a role in recruiting PcG complexes to this transgene. *lacZ* silencing is also relieved in a *ph* mutant (Fig 6G). These data show that the small Pho/Ph peaks can act as PREs in this transgene and suggest they also act as PREs within the *inv-en* domain.

We examined H3K27me3 accumulation over a transgene that had a 2.6kb fragment of *en* DNA, from -2.4kb to +139bp (including the known PREs and the *en* promoter) fused to *lacZ* and cloned into *pCaSpeR* with the mini-*white* reporter (S3B Fig, [37]). A small H3K27me3 domain formed over the *lacZ* gene in this transgene (S3C Fig). Like the signal over *P[7*.*5en-lacZ]* (Fig 6C), the highest level of H3K27me3 was found closest to the *en* fragment, and its intensity rapidly dropped about 50% over the ~2.5kb region of *lacZ* gene. When the fragment containing the known PREs was removed from this transgene (via FRT and loxP sites) [37], H3K27me3 was completely lost, even though *en* promoter remained (S3C Fig) [52]. The *en* sequences remaining in this transgene included 400bp upstream of the promoter and 139bp of the untranslated leader; analogous to the *en* promoter remaining in *en*^*∆1*.*5*^ (that deletes sequences from -1956 to -412bp). Thus, the *en* promoter alone is not sufficient to establish an H3K27me3 domain. We also examined the effect of an individual weak Pho/Ph peak (2R:7429281..7432610) on H3K27me3 accumulation in the *P[en-lacZ]* (S3D Fig). This fragment was able to incorporate H3K27me3 over *lacZ* in two different insertion sites in the genome (S3D Fig). This construct contains no embryo or imaginal disc enhancer [32], thus we could not access *lacZ* expression in these lines. We do note however, that three out of four *P[3*.*3en-lacZ]* exhibit pairing sensitive silencing, strongly suggesting this transgene contains a PRE. Finally to address the question of the relative strength of these presumed PREs, we inserted fragments with either strong or weak Pho/Ph peaks into a *ΦC31-white* reporter gene construct and integrated them in the *attP40* site; we also integrated vector alone at the *attP40* site. The vector alone inserted at this site gave transgenic flies with red eyes. Transgenic flies containing constructs with PREs from *en* or *Ubx* had an orange eye color, consistent with repression of *white* by the PRE. In contrast, transgenic flies with constructs with weak Pho/Ph peaks had a red eye color, similar to the vector alone (S3E Fig). We further cloned three weak peak fragments together in the reporter construct; even this was not able to repress the *white* gene (S3E Fig, middle picture). We note that at this chromosomal location we did not observe any PSS. In addition, the *en* and *Ubx* PREs were able to suppress the *yellow* gene present at the *ΦC31* landing site, while constructs with the weak peaks were not. These data show that the known PREs with the large Ph/Pho peaks are stronger silencers than the weak Ph/Pho binding sites in this assay.

## Discussion

Global identification of genomic sites to which PcG complexes bind and understanding the mechanism that controls expression of target genes by these complexes is essential to fathom how the PcG system regulates transcription and genome organization. The key conclusions that can be drawn from our experiments are: 1. Strong PREs of the *inv-en* domain are non-essential in the laboratory. 2. The size of the weak Pho and Ph peaks are not changed in the absence of the known PREs. 3. Weak Pho and Ph peaks interact with the *en* transcription unit 4. Known PREs are dispensable for maintenance of 3D structure of the local PcG domain. 5. A DNA fragment with weak Ph/Pho peaks can create a PcG domain in a transgene. These data change our view of PcG-recruitment to the *inv-en* domain; rather than having just a few strong PREs, there are many DNA fragments that can act together to recruit PcG proteins. As explained below, it is likely that our results can be generalized to many PcG targets.

### Evidence for weak PREs from other studies

PREs were identified as DNA fragments that could recruit PcG proteins to transgenes [15, 17], maintain expression of a reporter gene throughout development, and cause the transgenes to be derepressed in a PcG-mutant. Early chromatin-immunoprecipitation experiments showed that PREs are binding sites for PcG proteins [53], and genome-wide studies identified the largest PcG peaks at known PREs. Most PcG-target genes have only a few large PcG-protein peaks, often located near the promoter. However, there were indications from early transgenic studies that more than just a few PREs exist in PcG targets. For example, when testing fragments of *Ubx* DNA for regulatory activity in embryos, Müller and Bienz (1991) reported that two discrete DNA fragments could confer PcG-dependent restricted expression to a *bxd-Ubx-lacZ* reporter gene; one they called PBX and another called ABX. The *bxd* enhancer causes *bxd-Ubx-lacZ* to be expressed from the head to the tail of the embryo [parasegments (ps) 2–14], but is silenced in the anterior part of the embryo in the presence of either the PBX or the ABX fragment due to repression by the PcG-proteins. Notably the strength of repression by the PBX fragment was stronger than that by the ABX fragment suggesting the presence of strong and weak PREs.

The *bxd* enhancer is ‘primed’ to be PcG-regulated. In 1995, Jürg Müller used the UAS-Gal4 system to show that transient expression of Gal4-Pc could silence a *UAS-bxd-lacZ* transgene throughout embryogenesis, and showed that this silencing was dependent on endogenous PcG-genes [54]. This data suggests that once the *bxd*-fragment encounters PcG-proteins, it can maintain repression, essentially acting as a PRE. In contrast, a *UAS-NP6-lacZ* construct, containing the synthetic enhancer NP6, was only transiently silenced by Gal4-Pc; once Gal4-Pc was gone, the transgene was expressed again. Thus, on the *UAS-bxd-lacZ* transgene there was a kind of “handing off” from the Gal4-Pc to the endogenous PcG-proteins and this did not happen on the *UAS-NP6-lacZ* construct. Interestingly, the *bxd* enhancer contains a weak PcG-protein peak but on its own does not act as a PRE in transgenes (S4 Fig). We suggest that regulatory DNA from PcG-target genes is primed to be PcG-regulated and facilitates PcG-recruitment and H3K27me3 spreading.

### PcG-recruitment to *inv-en*

Examination of H3K27me3 levels in early embryos showed that H3K27me3 accumulates first over the *inv-en* known PREs and subsequently spreads to the rest of *inv*-*en* region [55]. It is therefore surprising that deletion of the known PREs does not lead to de-repression of En/Inv expression and therefore to major developmental defects and lethality. Perhaps even more surprising is that, apart from the DNA flanking the known *en* PREs, H3K27me3 levels are not decreased in the *inv*^*∆24*^ *en*^*∆1*.*5*^ larvae and the 3D structure of the *inv-en* domain is not disrupted by the loss of the known PREs. Clearly, recruitment of PcG proteins is highly redundant at the *inv*-*en* domain. Another surprising result in our experiments is the presence of the Ph protein at the *en* promoter in the absence of the promoter-proximal *en* PREs. We suggest that Ph, or other PRC1 components are recruited to the *en* promoter either by direct interaction with other transcription factors (i.e. Adf1, Cg) [56, 57] or via interaction with paused polymerase. It is interesting to note that in early studies, general transcription factors co-purified with PRC1 components [58].

### Why so many PREs in a PcG domain?

**Fig 7:**
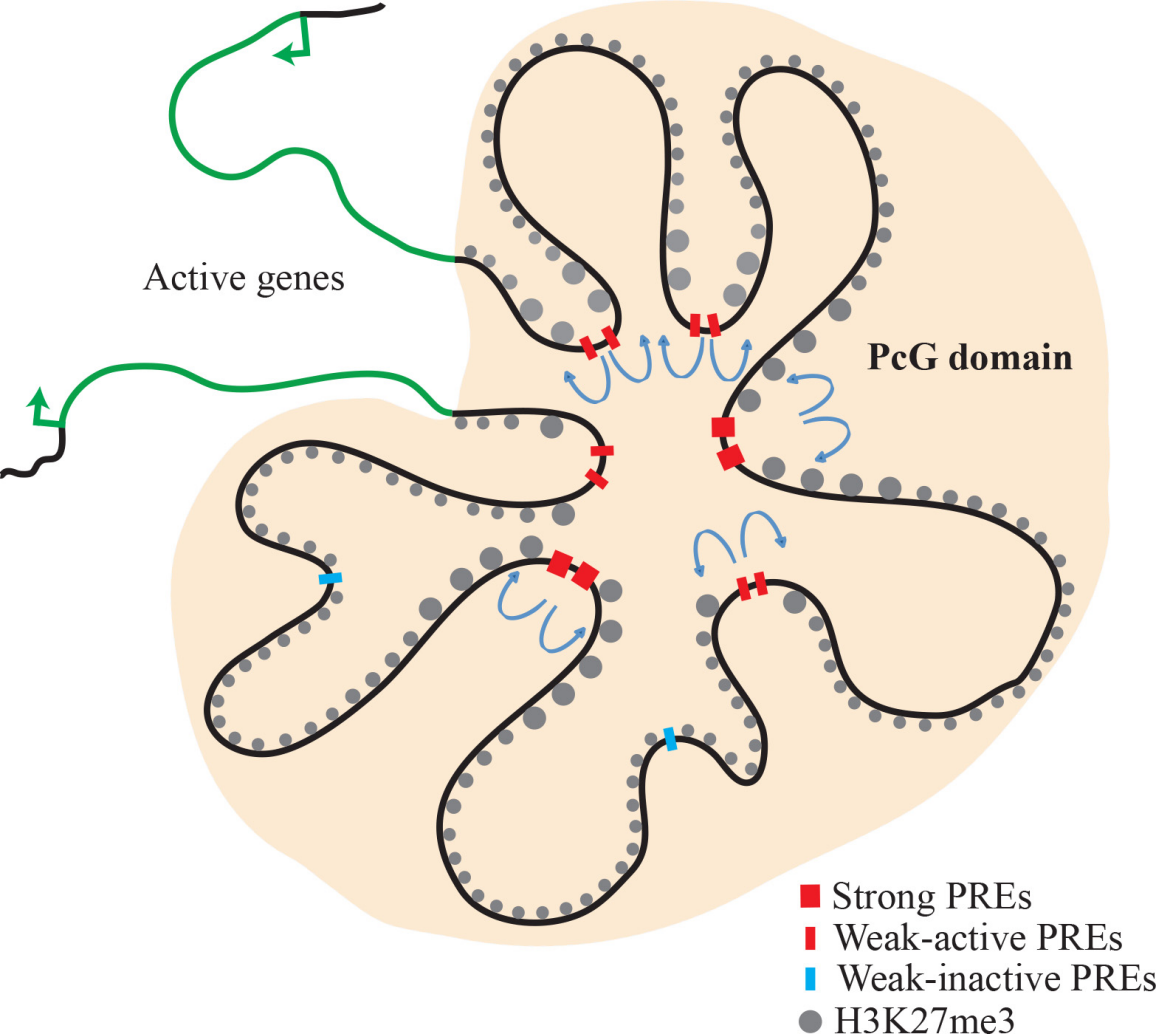
Strong and weak PREs act cooperatively for domain stabilization and H3K27me3 spreading. A cartoon of a PcG domain is shown. Within the PcG domain, repressed chromatin is shown by black line. H3K27me3 (grey spheres), weak-active PREs (red lines), weak-inactive PREs (blue lines), and strong PREs (red boxes) are also shown inside the domain. Weak-inactive PREs can be functional in some other tissue. Active genes are represented with green line and transcription start sites are represented with green arrows.

PcG recruitment and repression is a dynamic process; PREs recruit PcG complexes and they are anchored to PREs, but interactions between PRC1 and PRC2 components and the chromatin modifications imparted by them reinforce each other to stabilize and propagate the complexes [50, 59]. ChIP-experiments show that the enzymatic component of PRC2, E(z) is concentrated over the PREs, and the levels of H3K27me3 are very high flanking the strong PREs of *en* (Fig 2), suggesting that E(z) acts best on flanking nucleosomes and that the level of activity decreases with distance from the PRE. How far does the H3K27me3 domain spread from a PRE? Interestingly, the size of the H3K27me3 domain on our *en-lacZ* transgenes was relatively small, dropping rapidly throughout the 2.5kb of *lacZ* gene, suggesting that E(z) acts on a relatively local area. Given previous results showing the extreme sensitivity of PRE-transgenes to the position of insertion in the genome, we suspect that the degree of spreading will be dependent on the exact construct tested and the chromosomal insertion site. Nevertheless, our studies showed a remarkable consistency between the spreading of H3K27me3 through the *lacZ* gene for three different *en-lacZ* transgenes inserted at 5 different chromosomal locations. Thus we suggest that spreading from a single PRE is likely limited and that the weak Ph/Pho peaks facilitate spreading of the H3K27me3 mark both by direct recruitment of PcG proteins and by facilitating interactions with PcG proteins bound to other PREs (Fig 7). We note that the strong PREs are present in all cell types and tissues whereas weak peaks may be either stage or tissue specific, although at present we do not know what imparts this specificity on a PRE (Fig 7).

While PREs have been known for many years in Drosophila, identification of PREs in mammalian PcG-targets has been more difficult in part because very high PcG-binding peaks are not present in mammalian PcG-target genes, although many small PcG-binding peaks do exist. Interestingly, a detailed analysis of PcG recruitment to the *HoxD* locus in mice suggests a large number of binding sites cooperate to recruit PRC2 [60]. These authors show that deletions within the 300kb *HoxD* locus do not disrupt the H3K27me3 domain, suggesting the presence of multiple PRC2-recruitment sites. Interestingly, previous results on single PRE deletions in the endogenous *Ubx* and *Abd-B* genes showed only minimal mis-expression of these genes and weak phenotypes, suggesting at least partial redundancy in PcG recruitment at these loci [29–31, 61]. The consequences of these PRE deletions on the H3K27me3 domain were not examined. Our data suggest that multiple, weak PREs can recruit PcG proteins to the *inv-en* domain in the absence of the strong PREs. We suggest that PcG recruitment in Drosophila is similar to that in mammals, except that in mammals, PcG-target genes lack strong PREs.

## Materials and Methods

### Fly strains

Generation of *en*^*Δ1*.*5*^ and *inv*^*Δ24*^ mutants have been described [32, 42]. The double mutant *inv*^*Δ24*^-*en*^*Δ1*.*5*^ was obtained using P-element mediated male recombination [62]. The deletions were validated both by PCR and by the absence of those sequences in ChIP-seq data. All flies were kept at 25°C. Generation of constructs and transgenic lines has been discussed previously [32].

### qRT-PCR

Total RNA was collected from 20 third instar larvae wing imaginal disks using Trizol (Invitrogen) according to manufacturer’s protocol. One-step RT-qPCR was performed with the SensiFAST SYBR No-ROX One-Step kit (Bioline) on a Roche Lightcycler 480 according to manufacturer instructions [63]. Primers used are listed in S2 Table.

### Staining of imaginal disks

For antibody staining, imaginal disks and brains were collected from third instar larvae and fixed for 23 min [0.1 M PIPES (pH 6.9), 1 mM EGTA (pH 6.9), 1.0% Triton X-100, 2mM MgSO4, 4% formaldehyde (Ted Pella, Inc.)]. Fixed tissues were blocked for 2 hrs at 4°C with blocking buffer (1X PBS, 0.1% Tween-20 and 5% normal goat serum). Tissues were incubated over night at 4°C with primary antibody diluted in blocking buffer (rabbit anti-En 1:500 dilution, Santa Cruz Biotechnology, Inc.; mouse anti-ßgal 1:500 dilution, Invitrogen). After removal of primary antibody, tissues were washed (1X PBS, 0.1% Tween-20) four times- 15 min each. Washed tissues were incubated in fluorescent tagged secondary antibody diluted in blocking buffer for 2 hrs at 4°C in dark, washed 3 times and mounted with DAPI-Vectashield (Vector Laboratories). All wing imaginal discs were quantified by ImageJ.

### ChIP and ChIP-qPCR

Protocol for carrying out ChIP in larval tissue has been described previously [63], with minor changes: fixed brains and imaginal discs were dissected from 10 third instar larvae, and before incubating the sonicated chromatin with antibodies, 3.3% of each sample was saved for input reactions. ChIP was performed with 1:100 dilutions of anti-Pho [64] and anti-Ph antibodies (a kind gift from Donna Arndt-Jovin) and 1:200 dilutions of anti-H3K27me3 (Millipore, 17–622) antibodies. Quantitative PCR on ChIP samples was performed as described previously [26]. Lightcycler 480 Real-Time PCR System (Roche Applied Science) and Lightcycler 480 DNA SYBR Green I Master Mix (Roche Applied Science) were used for performing ChIP-qPCR.

### ChIP-seq and data analysis

Following purification of immunoprecipitated DNA, Illumina libraries were prepared using TruSeq DNA Sample Prep Kit V2 as described (http://ethanomics.wordpress.com/chip-seq-library-construction-using-the-illumina-truseq-adapters/). Peak calling for Pho was conducted using MACS v1.4.2 [65]. All ChIP-seq data sets were aligned using Casava (version 1.8) to the Drosophila reference genome (release 5.22/6.02). All ChIP-seq experiments were performed with 2 biological replicates yielding similar results. Data was normalized as reads per million except the embryo Ph and H3K27me3 data in Fig 1, normalization of this data is explained in Bowman et al. (Bowman *et al*., 2014 [38]). The same H3K27me3 data was done in several figures (1, 2D, 3A, 5A). Peak calling for Pho was conducted using MACS v1.4.2 [65]. Shifting model building for prediction of fragment length was turned off (—nomodel) and a fixed standard background model for peak calling was used to increase sensitivity (—nolambda). In addition, we specified a p-value cutoff of 0.00001 for the output peaks. For in depth analysis of the binding regions, output peaks were split with Peaksplitter v0.1 [40] and ~20% (peak height≥ 50) of the split peaks were considered for further analysis to rank them into null, weak and strong binding sites. Sequencing data have been deposited to the Gene Expression Omnibus.

### 4C-seq

Chromosome conformation capture (3C) protocol was followed as described by Tolhuis *et al*. [66] with minor modifications. To generate 4C-library, we followed the protocol described by Tolhuis *et al*. [67]. Brains and disks from 10 third instar larvae were fixed using freshly prepared fixing solution containing 2% formaldehyde and 1X PBS prepared from 16% formaldehyde (Ted Pella, Inc.). Fixation reaction was quenched using 125 mM Glycine. After fine dissection and nuclei isolation, nuclei pellet was dissolved in 1X NEB buffer 3. After the SDS treatment the chromatin was digested overnight with 5 μl EcoRI (200 U, NEB). Next day, after inactivating EcoRI, the digested chromatin was ligated for 6 hrs with 0.5 μl ligase (200 U, NEB) in 2ml of ligation reaction. The ligated library was purified as stated in the reference mentioned above. Finally the library was dissolved in 400 μl ultrapure water or 10 mM Tris-HCl (pH 8.0).

100 μl of the 3C library was digested with 5 μl of DpnII (50 U, NEB). After the enzyme was inactivated, digested chromatin was re-ligated using 5 μl of ligase (200 U, NEB) in a total volume of 2 ml ligation reaction to promote self-ligation events. Finally purification of the library was carried out. Inverse PCR was carried out using 8 different custom made reverse primers (containing unique 5 nucleotide bar code) and one universal forward primer to produce Illumina-4C-seq libraries. The primer sequence used for 4C-seq library amplification is mentioned in S2 Table. PCR amplification was carried out using Kapa HiFi HotStart Ready Mix (KAPA Biosystems, cat no. KK2601) with 90 ng of DNA and 2.5 μl of forward primer (10mM) and 1.7 μl of reverse primer (10mM). Amplification program in thermal cycler was: denaturation- 95°C for 30 sec, amplification- 25 cycles of 98°C for 10 sec, 64°C for 30 sec and 72°C for 30 sec; final elongation step was 72°C for 1 min. After pooling together 6 PCR reactions, it was purified using High Pure PCR Product Purification kit (Roche, cat no. 11732676001). Finally 4C-PCR products from WT and mutant lines were combined in preferred ratios for sequencing.

### 4C-data analysis

Procedure to detect significant interactions (peaks) in 4C was adapted from Tolhuis *et al*. and Ghavi-Helm *et al*. [51, 67]. Read counts were transformed using variance stabilizing transformation from the DESeq2 package in R [68] and then transformed counts from chr2R (chromosome with bait fragment) were smoothed using loess. Region immediately surrounding bait fragment was excluded from fit (chr2R:7410000..7416000). Interactions with p-value < 0.01 in both replicates and FDR < 0.1 in at least one replicate were selected as bona-fide interactions.

Reads from all the NGS data were deposited in the NCBI-GEO under accession numbers GSE77342.

## Supporting Information

S1 Fig Bioinformatic categorization of Pho peaks. (A) ChIP-seq profiles of H3K27me3 (top row) and Pho (second row) in WT over the *inv-en* domain. MACS identified Pho peaks after (third row) and before (fourth row) using Peaksplitter are indicated by navy blue boxes. (B) Coordinates, peak heights and ranks of all the split Pho peaks from the *inv-en* domain. Pho peaks associated with characterized PREs are shaded with red and weak Pho peaks are shaded with blue. (C) Categorization of the top 20% of the split Pho peaks according to their heights. The Pho Peaks from (B) are shown as dots and indicated by color. Note that one of the characterized PREs has a small Pho peak (black circle surrounding the red dot).
(TIF)

S2 Fig Removal of characterized PREs leads to a defect in fusion of the adult abdominal cuticle. (A) Female (upper panel) and male (lower panel) WT, *en*^*∆1*.*5*^, *inv*^*∆24*^ and *inv*^*∆24*^ *en*^*∆1*.*5*^ adults. Defect in fly abdominal cuticle is indicated by red arrows. (B) Schematic of the pBac[WH]f06870 present at the *inv*^*∆24*^ deletion site. *inv*^*∆24*^ was made by recombination between two P[WH] elements, leaving an intact element at the site of the deletion [32]. (C) Quantification of total transcripts of *E(Pc)*, *inv*, *en* and *tou* in WT and *inv*^*∆24*^*en*^*∆1*.*5*^ relative to total RpL32 transcript in larval wing imaginal disks; data presented is from the average of two independent biological samples with three replicates each (mean±SEM). (D) Quantification of the En expressing area over the total wing disc area, data was collected from 20 imaginal discs of WT and *inv*^*∆24*^ *en*^*∆1*.*5*^ (mean±SEM). n.s. not significant by student T test. (E) Quantification of fluorescent pixel intensity in the En expressing region in the Wing disc, in the indicated genotypes, data was collected from 15 imaginal discs of WT and *inv*^*∆24*^ *en*^*∆1*.*5*^ (mean±SEM). (F) Total PolII accumulation quantified on promoters and body of *E(Pc)*, *inv*, *en* and *tou*. The *rpl32* gene is used as a positive control. Results are shown as fold enrichment over background signal and are the average of two independent biological samples with three replicates each (mean±SEM).
(TIF)

S3 Fig Fragments associated with weak peaks incorporate H3K27me3 over a transgene. (A) Quantification of E(z) on *P[7*.*5en-lacZ]* at the *en* Promoter-7.5 junction and at the 5’ end of *lacZ* gene. *en*PRE2 and PRE_D_ (a *Ubx* PRE) were used as positive control for this experiment. Results are shown as fold enrichment over control signal and are the average of two independent biological samples with three replicates each (mean±SEM). (B) Diagram of the P-element based transgenic construct with and without *en* characterized PREs [37]. (C) Quantification of H3K27me3 over *lacZ*, gene. Regions amplified by qPCR are indicated over *lacZ* gene in the diagram; fragments from *e(Pc)* and *tou* were used as negative controls. Results are shown as fold enrichment over background signal and are the average of two independent biological samples with three replicates each (mean±SEM). Statistical analysis of differential H3K27me3 accumulation was performed using Student’s t-test, P-values ≤ 0.05. Only the significant differences with WT are shown with ‘*’. (D) Schematic of a transgene containing a fragment associated with a single weak peak (2R:7429281..7432610, fragment K from Cheng et al., 2014 [32]) is shown in upper panel; quantification of H3K27me3 over *lacZ* in two transgenic lines containing above transgene is shown. Results are shown as fold enrichment over control signal and are the average of two independent biological samples with three replicates each (mean±SEM). (E) Schematic of the *attP40* site after transgene insertion is shown in the top panel, inserted vector is shown in green color. Pictures of the fly eye containing either the strong and weak PcG peaks are shown in the bottom panel. Fragment coordinates used in this assay are also shown. Construct ‘*attB-P[acman]-Ap*^*R*^*’* [69] was modified by the addition of an eye enhancer from the *white* gene [32]. Note also that the body color of the enPREs and PRE_D_ lines is yellow whereas the other lines have a darker body color. This lighter body color is the result of repression of the *y+* transgene present at the attP40 site.
(TIF)

S4 Fig Strong and weak peaks are present at Bithorax and Antennapedia domain. (A) ChIP-seq profiles of Pho, Ph (top two rows) and of H3K27me3 (bottom row) in WT over the Bithorax complex. Well-characterized PREs are indicated with blue arrowheads. Positions of the genes (navy blue) are shown at the very bottom. Regulatory fragments (with corresponding coordinates) identified by Müller and Bienz (1991) [16] are shown in the figure. (B) Profiles of the same over *Antp* domain.
(TIF)

S1 Table List of Pho, Zeste, Spps, Dsp1 and Grh consensus binding sites and the GTGT and GAGA motifs found within 1kb region encompassing the small peaks. The center of the peak was identified and 500bp upstream and downstream were chosen for this analysis. The coordinates of the small peaks are shown in the left column.
(TIFF)

S2 Table List of primers used. (XLSX)

## References

[1] Lewis EB. A gene complex controlling segmentation in Drosophila. Nature. 1978;276(5688):565–70. . 103000 10.1038/276565a0

[2] Di Croce L, Helin K. Transcriptional regulation by Polycomb group proteins. Nature structural & molecular biology. 2013;20(10):1147–55. 10.1038/nsmb.2669 . 24096405

[3] Simon JA, Kingston RE. Occupying chromatin: Polycomb mechanisms for getting to genomic targets, stopping transcriptional traffic, and staying put. Molecular cell. 2013;49(5):808–24. 10.1016/j.molcel.2013.02.013 23473600 PMC3628831

[4] Shao Z, Raible F, Mollaaghababa R, Guyon JR, Wu CT, Bender W, et al Stabilization of chromatin structure by PRC1, a Polycomb complex. Cell. 1999;98(1):37–46. 10.1016/S0092-8674(00)80604-2 . 10412979

[5] Kassis JA, Brown JL. Polycomb group response elements in Drosophila and vertebrates. Advances in genetics. 2013;81:83–118. 10.1016/B978-0-12-407677-8.00003–8 23419717 PMC4157523

[6] Sing A, Pannell D, Karaiskakis A, Sturgeon K, Djabali M, Ellis J, et al A vertebrate Polycomb response element governs segmentation of the posterior hindbrain. Cell. 2009;138(5):885–97. 10.1016/j.cell.2009.08.020 . 19737517

[7] Woo CJ, Kharchenko PV, Daheron L, Park PJ, Kingston RE. A region of the human HOXD cluster that confers Polycomb-group responsiveness. Cell. 2010;140(1):99–110. 10.1016/j.cell.2009.12.022 20085705 PMC3324942

[8] Basu A, Dasari V, Mishra RK, Khosla S. The CpG island encompassing the promoter and first exon of human DNMT3L gene is a PcG/TrX response element (PRE). PloS one. 2014;9(4):e93561 10.1371/journal.pone.0093561 24743422 PMC3990577

[9] Mendenhall EM, Koche RP, Truong T, Zhou VW, Issac B, Chi AS, et al GC-rich sequence elements recruit PRC2 in mammalian ES cells. PLoS genetics. 2010;6(12):e1001244 10.1371/journal.pgen.1001244 21170310 PMC3000368

[10] Lynch MD, Smith AJ, De Gobbi M, Flenley M, Hughes JR, Vernimmen D, et al An interspecies analysis reveals a key role for unmethylated CpG dinucleotides in vertebrate Polycomb complex recruitment. The EMBO journal. 2012;31(2):317–29. 10.1038/emboj.2011.399 22056776 PMC3261560

[11] Martin N, Popov N, Aguilo F, O'Loghlen A, Raguz S, Snijders AP, et al Interplay between Homeobox proteins and Polycomb repressive complexes in p16INK(4)a regulation. The EMBO journal. 2013;32(7):982–95. 10.1038/emboj.2013.37 23455154 PMC3616285

[12] Ren X, Kerppola TK. REST interacts with Cbx proteins and regulates polycomb repressive complex 1 occupancy at RE1 elements. Molecular and cellular biology. 2011;31(10):2100–10. 10.1128/MCB.05088-11 21402785 PMC3133345

[13] Dietrich N, Lerdrup M, Landt E, Agrawal-Singh S, Bak M, Tommerup N, et al REST-mediated recruitment of polycomb repressor complexes in mammalian cells. PLoS genetics. 2012;8(3):e1002494 10.1371/journal.pgen.1002494 22396653 PMC3291536

[14] Chan CS, Rastelli L, Pirrotta V. A Polycomb response element in the Ubx gene that determines an epigenetically inherited state of repression. The EMBO journal. 1994;13(11):2553–64. 7912192 10.1002/j.1460-2075.1994.tb06545.x PMC395129

[15] Chiang A, O'Connor MB, Paro R, Simon J, Bender W. Discrete Polycomb-binding sites in each parasegmental domain of the bithorax complex. Development. 1995;121(6):1681–9. . 7600985 10.1242/dev.121.6.1681

[16] Muller J, Bienz M. Long range repression conferring boundaries of Ultrabithorax expression in the Drosophila embryo. The EMBO journal. 1991;10(11):3147–55. 1680676 10.1002/j.1460-2075.1991.tb04876.x PMC453036

[17] Zink B, Engstrom Y, Gehring WJ, Paro R. Direct interaction of the Polycomb protein with Antennapedia regulatory sequences in polytene chromosomes of Drosophila melanogaster. The EMBO journal. 1991;10(1):153–62. 1671215 10.1002/j.1460-2075.1991.tb07931.x PMC452623

[18] Simon J, Chiang A, Bender W, Shimell MJ, O'Connor M. Elements of the Drosophila bithorax complex that mediate repression by Polycomb group products. Developmental biology. 1993;158(1):131–44. 10.1006/dbio.1993.1174 . 8101171

[19] Kassis JA. Unusual properties of regulatory DNA from the Drosophila engrailed gene: three "pairing-sensitive" sites within a 1.6-kb region. Genetics. 1994;136(3):1025–38. 8005412 10.1093/genetics/136.3.1025 PMC1205860

[20] Kassis JA, VanSickle EP, Sensabaugh SM. A fragment of engrailed regulatory DNA can mediate transvection of the white gene in Drosophila. Genetics. 1991;128(4):751–61. 1655566 10.1093/genetics/128.4.751 PMC1204549

[21] Lanzuolo C, Roure V, Dekker J, Bantignies F, Orlando V. Polycomb response elements mediate the formation of chromosome higher-order structures in the bithorax complex. Nature cell biology. 2007;9(10):1167–74. 10.1038/ncb1637 . 17828248

[22] Bantignies F, Roure V, Comet I, Leblanc B, Schuettengruber B, Bonnet J, et al Polycomb-dependent regulatory contacts between distant Hox loci in Drosophila. Cell. 2011;144(2):214–26. 10.1016/j.cell.2010.12.026 . 21241892

[23] Vazquez J, Muller M, Pirrotta V, Sedat JW. The Mcp element mediates stable long-range chromosome-chromosome interactions in Drosophila. Molecular biology of the cell. 2006;17(5):2158–65. 10.1091/mbc.E06-01-0049 16495335 PMC1446072

[24] Bantignies F, Grimaud C, Lavrov S, Gabut M, Cavalli G. Inheritance of Polycomb-dependent chromosomal interactions in Drosophila. Genes & development. 2003;17(19):2406–20. 10.1101/gad.269503 14522946 PMC218078

[25] Li HB, Muller M, Bahechar IA, Kyrchanova O, Ohno K, Georgiev P, et al Insulators, not Polycomb response elements, are required for long-range interactions between Polycomb targets in Drosophila melanogaster. Molecular and cellular biology. 2011;31(4):616–25. 10.1128/MCB.00849-10 21135119 PMC3028641

[26] Brown JL, Kassis JA. Architectural and functional diversity of polycomb group response elements in Drosophila. Genetics. 2013;195(2):407–19. 10.1534/genetics.113.153247 23934890 PMC3781969

[27] Brown JL, Mucci D, Whiteley M, Dirksen ML, Kassis JA. The Drosophila Polycomb group gene pleiohomeotic encodes a DNA binding protein with homology to the transcription factor YY1. Molecular cell. 1998;1(7):1057–64. . 9651589 10.1016/s1097-2765(00)80106-9

[28] Schwartz YB, Kahn TG, Nix DA, Li XY, Bourgon R, Biggin M, et al Genome-wide analysis of Polycomb targets in Drosophila melanogaster. Nature genetics. 2006;38(6):700–5. 10.1038/ng1817 . 16732288

[29] Mihaly J, Hogga I, Gausz J, Gyurkovics H, Karch F. In situ dissection of the Fab-7 region of the bithorax complex into a chromatin domain boundary and a Polycomb-response element. Development. 1997;124(9):1809–20. . 9165128 10.1242/dev.124.9.1809

[30] Mihaly J, Barges S, Sipos L, Maeda R, Cleard F, Hogga I, et al Dissecting the regulatory landscape of the Abd-B gene of the bithorax complex. Development. 2006;133(15):2983–93. 10.1242/dev.02451 . 16818450

[31] Sipos L, Kozma G, Molnar E, Bender W. In situ dissection of a Polycomb response element in Drosophila melanogaster. Proceedings of the National Academy of Sciences of the United States of America. 2007;104(30):12416–21. 10.1073/pnas.0703144104 17640916 PMC1941339

[32] Cheng Y, Brunner AL, Kremer S, DeVido SK, Stefaniuk CM, Kassis JA. Co-regulation of invected and engrailed by a complex array of regulatory sequences in Drosophila. Developmental biology. 2014;395:131–43. 10.1016/j.ydbio.2014.08.021 . 25172431 PMC4189978

[33] Negre N, Hennetin J, Sun LV, Lavrov S, Bellis M, White KP, et al Chromosomal distribution of PcG proteins during Drosophila development. PLoS biology. 2006;4(6):e170 10.1371/journal.pbio.0040170 16613483 PMC1440717

[34] Cunningham MD, Brown JL, Kassis JA. Characterization of the Polycomb group response elements of the Drosophila melanogaster invected Locus. Molecular and cellular biology. 2010;30(3):820–8. 10.1128/MCB.01287-09 19948883 PMC2812238

[35] Gustavson E, Goldsborough AS, Ali Z, Kornberg TB. The Drosophila engrailed and invected genes: partners in regulation, expression and function. Genetics. 1996;142(3):893–906. 8849895 10.1093/genetics/142.3.893 PMC1207026

[36] Celniker SE, Dillon LA, Gerstein MB, Gunsalus KC, Henikoff S, Karpen GH, et al Unlocking the secrets of the genome. Nature. 2009;459(7249):927–30. 10.1038/459927a 19536255 PMC2843545

[37] DeVido SK, Kwon D, Brown JL, Kassis JA. The role of Polycomb-group response elements in regulation of engrailed transcription in Drosophila. Development. 2008;135(4):669–76. 10.1242/dev.014779 . 18199580

[38] Bowman SK, Deaton AM, Domingues H, Wang PI, Sadreyev RI, Kingston RE, et al H3K27 modifications define segmental regulatory domains in the Drosophila bithorax complex. eLife. 2014;3:e02833 10.7554/eLife.02833 25082344 PMC4139060

[39] Americo J, Whiteley M, Brown JL, Fujioka M, Jaynes JB, Kassis JA. A complex array of DNA-binding proteins required for pairing-sensitive silencing by a Polycomb group response element from the Drosophila engrailed gene. Genetics. 2002;160(4):1561–71. 11973310 10.1093/genetics/160.4.1561 PMC1462036

[40] Salmon-Divon M, Dvinge H, Tammoja K, Bertone P. PeakAnalyzer: genome-wide annotation of chromatin binding and modification loci. BMC bioinformatics. 2010;11:415 10.1186/1471-2105-11-415 20691053 PMC2923140

[41] Oktaba K, Gutierrez L, Gagneur J, Girardot C, Sengupta AK, Furlong EE, et al Dynamic regulation by polycomb group protein complexes controls pattern formation and the cell cycle in Drosophila. Developmental cell. 2008;15(6):877–89. 10.1016/j.devcel.2008.10.005 . 18993116

[42] Cheng Y, Kwon DY, Arai AL, Mucci D, Kassis JA. P-element homing is facilitated by engrailed Polycomb-group response elements in Drosophila melanogaster. PloS one. 2012;7(1):e30437 10.1371/journal.pone.0030437 22276200 PMC3261919

[43] Thibault ST, Singer MA, Miyazaki WY, Milash B, Dompe NA, Singh CM, et al A complementary transposon tool kit for Drosophila melanogaster using P and piggyBac. Nature genetics. 2004;36(3):283–7. 10.1038/ng1314 . 14981521

[44] Schaaf CA, Misulovin Z, Gause M, Koenig A, Gohara DW, Watson A, et al Cohesin and Polycomb proteins functionally interact to control transcription at silenced and active genes. PLoS genetics. 2013;9(6):e1003560 10.1371/journal.pgen.1003560 23818863 PMC3688520

[45] Kopp A, Duncan I. Anteroposterior patterning in adult abdominal segments of Drosophila. Developmental biology. 2002;242(1):15–30. 10.1006/dbio.2001.0529 . 11795937

[46] Enderle D, Beisel C, Stadler MB, Gerstung M, Athri P, Paro R. Polycomb preferentially targets stalled promoters of coding and noncoding transcripts. Genome research. 2011;21(2):216–26. 10.1101/gr.114348.110 21177970 PMC3032925

[47] Kwon D, Mucci D, Langlais KK, Americo JL, DeVido SK, Cheng Y, et al Enhancer-promoter communication at the Drosophila engrailed locus. Development. 2009;136(18):3067–75. 10.1242/dev.036426 19675130 PMC2730364

[48] Wang L, Brown JL, Cao R, Zhang Y, Kassis JA, Jones RS. Hierarchical recruitment of polycomb group silencing complexes. Molecular cell. 2004;14(5):637–46. 10.1016/j.molcel.2004.05.009 . 15175158

[49] Schuettengruber B, Oded Elkayam N, Sexton T, Entrevan M, Stern S, Thomas A, et al Cooperativity, specificity, and evolutionary stability of Polycomb targeting in Drosophila. Cell reports. 2014;9(1):219–33. 10.1016/j.celrep.2014.08.072 . 25284790

[50] Kahn TG, Stenberg P, Pirrotta V, Schwartz YB. Combinatorial interactions are required for the efficient recruitment of pho repressive complex (PhoRC) to Polycomb response elements. PLoS genetics. 2014;10(7):e1004495 10.1371/journal.pgen.1004495 25010632 PMC4091789

[51] Ghavi-Helm Y, Klein FA, Pakozdi T, Ciglar L, Noordermeer D, Huber W, et al Enhancer loops appear stable during development and are associated with paused polymerase. Nature. 2014;512(7512):96–100. 10.1038/nature13417 . 25043061

[52] Schuettengruber B, Cavalli G. Polycomb domain formation depends on short and long distance regulatory cues. PloS one. 2013;8(2):e56531 10.1371/journal.pone.0056531 23437158 PMC3577894

[53] Strutt H, Cavalli G, Paro R. Co-localization of Polycomb protein and GAGA factor on regulatory elements responsible for the maintenance of homeotic gene expression. The EMBO journal. 1997;16(12):3621–32. 10.1093/emboj/16.12.3621 9218803 PMC1169986

[54] Muller J. Transcriptional silencing by the Polycomb protein in Drosophila embryos. The EMBO journal. 1995;14(6):1209–20. 7720711 10.1002/j.1460-2075.1995.tb07104.x PMC398198

[55] Li XY, Harrison MM, Villalta JE, Kaplan T, Eisen MB. Establishment of regions of genomic activity during the Drosophila maternal to zygotic transition. eLife. 2014;3: e03737 10.7554/eLife.03737 25313869 PMC4358338

[56] Orsi GA, Kasinathan S, Hughes KT, Saminadin-Peter S, Henikoff S, Ahmad K. High-resolution mapping defines the cooperative architecture of Polycomb response elements. Genome research. 2014;24(5):809–20. 10.1101/gr.163642.113 24668908 PMC4009610

[57] Ray P, De S, Mitra A, Bezstarosti K, Demmers JA, Pfeifer K, et al Combgap contributes to recruitment of Polycomb group proteins in Drosophila. Proceedings of the National Academy of Sciences of the United States of America. 2016 10.1073/pnas.1520926113 27001825 PMC4833261

[58] Saurin AJ, Shao Z, Erdjument-Bromage H, Tempst P, Kingston RE. A Drosophila Polycomb group complex includes Zeste and dTAFII proteins. Nature. 2001;412(6847):655–60. 10.1038/35088096 . 11493925

[59] Kalb R, Latwiel S, Baymaz HI, Jansen PW, Muller CW, Vermeulen M, et al Histone H2A monoubiquitination promotes histone H3 methylation in Polycomb repression. Nature structural & molecular biology. 2014;21(6):569–71. 10.1038/nsmb.2833 . 24837194

[60] Schorderet P, Lonfat N, Darbellay F, Tschopp P, Gitto S, Soshnikova N, et al A genetic approach to the recruitment of PRC2 at the HoxD locus. PLoS genetics. 2013;9(11):e1003951 10.1371/journal.pgen.1003951 24244202 PMC3820793

[61] Kozma G, Bender W, Sipos L. Replacement of a Drosophila Polycomb response element core, and in situ analysis of its DNA motifs. Molecular genetics and genomics: MGG. 2008;279(6):595–603. 10.1007/s00438-008-0336-3 . 18350319

[62] Preston CR, Sved JA, Engels WR. Flanking duplications and deletions associated with P-induced male recombination in Drosophila. Genetics. 1996;144(4):1623–38. 8978050 10.1093/genetics/144.4.1623 PMC1207714

[63] Langlais KK, Brown JL, Kassis JA. Polycomb group proteins bind an engrailed PRE in both the "ON" and "OFF" transcriptional states of engrailed. PloS one. 2012;7(11):e48765 10.1371/journal.pone.0048765 23139817 PMC3490902

[64] Brown JL, Fritsch C, Mueller J, Kassis JA. The Drosophila pho-like gene encodes a YY1-related DNA binding protein that is redundant with pleiohomeotic in homeotic gene silencing. Development. 2003;130(2):285–94. . 12466196 10.1242/dev.00204

[65] Zhang Y, Liu T, Meyer CA, Eeckhoute J, Johnson DS, Bernstein BE, et al Model-based analysis of ChIP-Seq (MACS). Genome biology. 2008;9(9):R137 10.1186/gb-2008-9-9-r137 18798982 PMC2592715

[66] Tolhuis B, Blom M, Kerkhoven RM, Pagie L, Teunissen H, Nieuwland M, et al Interactions among Polycomb domains are guided by chromosome architecture. PLoS genetics. 2011;7(3):e1001343 10.1371/journal.pgen.1001343 21455484 PMC3063757

[67] Tolhuis B, Blom M, van Lohuizen M. Chromosome conformation capture on chip in single Drosophila melanogaster tissues. Methods. 2012;58(3):231–42. 10.1016/j.ymeth.2012.04.003 . 22525789

[68] Love MI, Huber W, Anders S. Moderated estimation of fold change and dispersion for RNA-seq data with DESeq2. Genome biology. 2014;15(12):550 10.1186/s13059-014-0550-8 25516281 PMC4302049

[69] Venken KJ, He Y, Hoskins RA, Bellen HJ. P[acman]: a BAC transgenic platform for targeted insertion of large DNA fragments in D. melanogaster. Science. 2006;314(5806):1747–51. 10.1126/science.1134426 . 17138868

