# Supplementary figures and images for: Formation of a Polycomb-Domain in the Absence of Strong Polycomb Response Elements
Source: PLoS Genet. 2016 Jul 28;12(7):e1006200. doi: 10.1371/journal.pgen.1006200 (PMC4965088; doi:10.1371/journal.pgen.1006200)

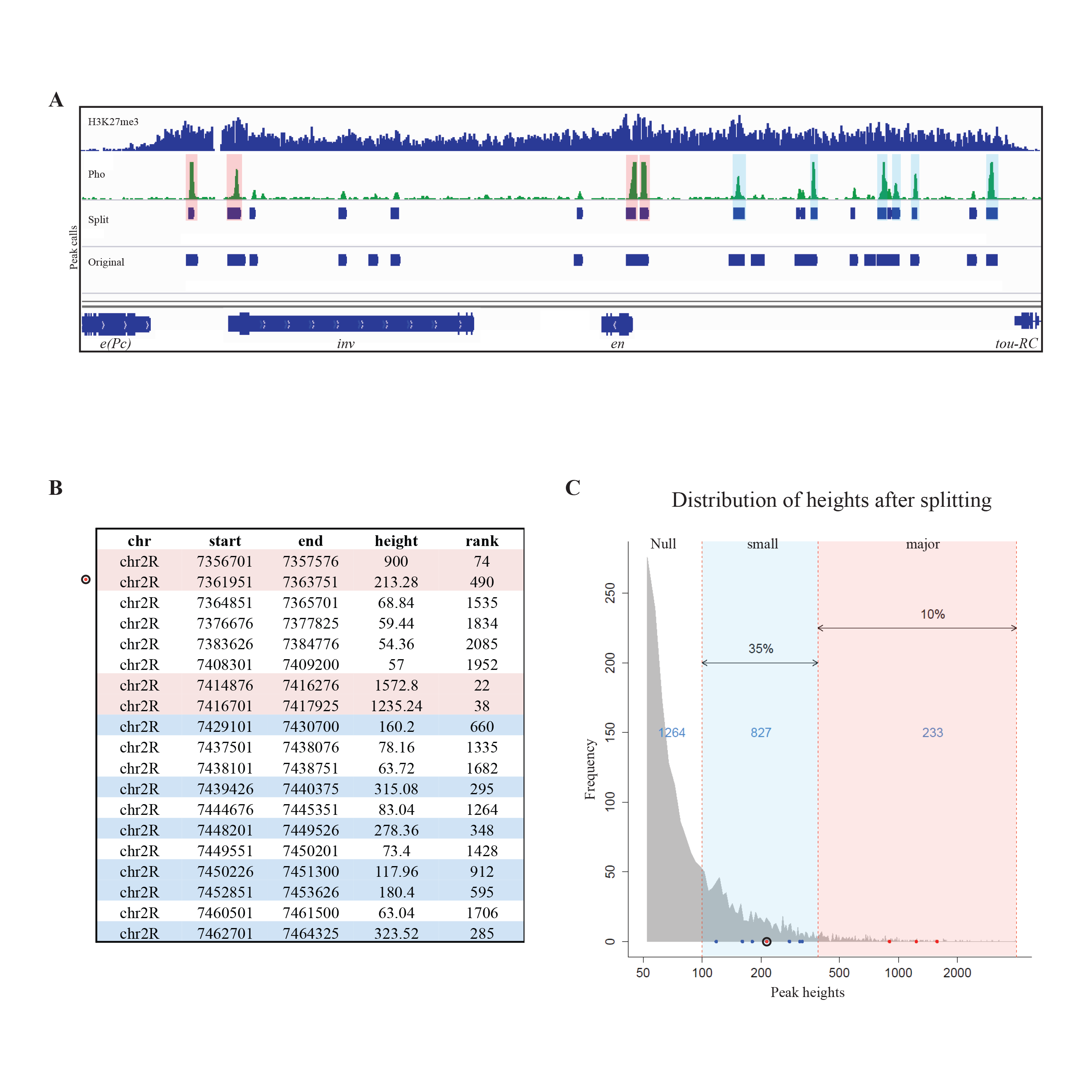

Supplement: S1 Fig — (A) ChIP-seq profiles of H3K27me3 (top row) and Pho (second row) in WT over the inv-en domain. MACS identified Pho peaks after (third row) and before (fourth row) using Peaksplitter are indicated by navy blue boxes. (B) Coordinates, peak heights and ranks of all the split Pho peaks from the inv-en domain. Pho peaks associated with characterized PREs are shaded with red and weak Pho peaks are shaded with blue. (C) Categorization of the top 20% of the split Pho peaks according to their heights. The Pho Peaks from (B) are shown as dots and indicated by color. Note that one of the characterized PREs has a small Pho peak (black circle surrounding the red dot). (TIF) [file pgen.1006200.s001.tif]

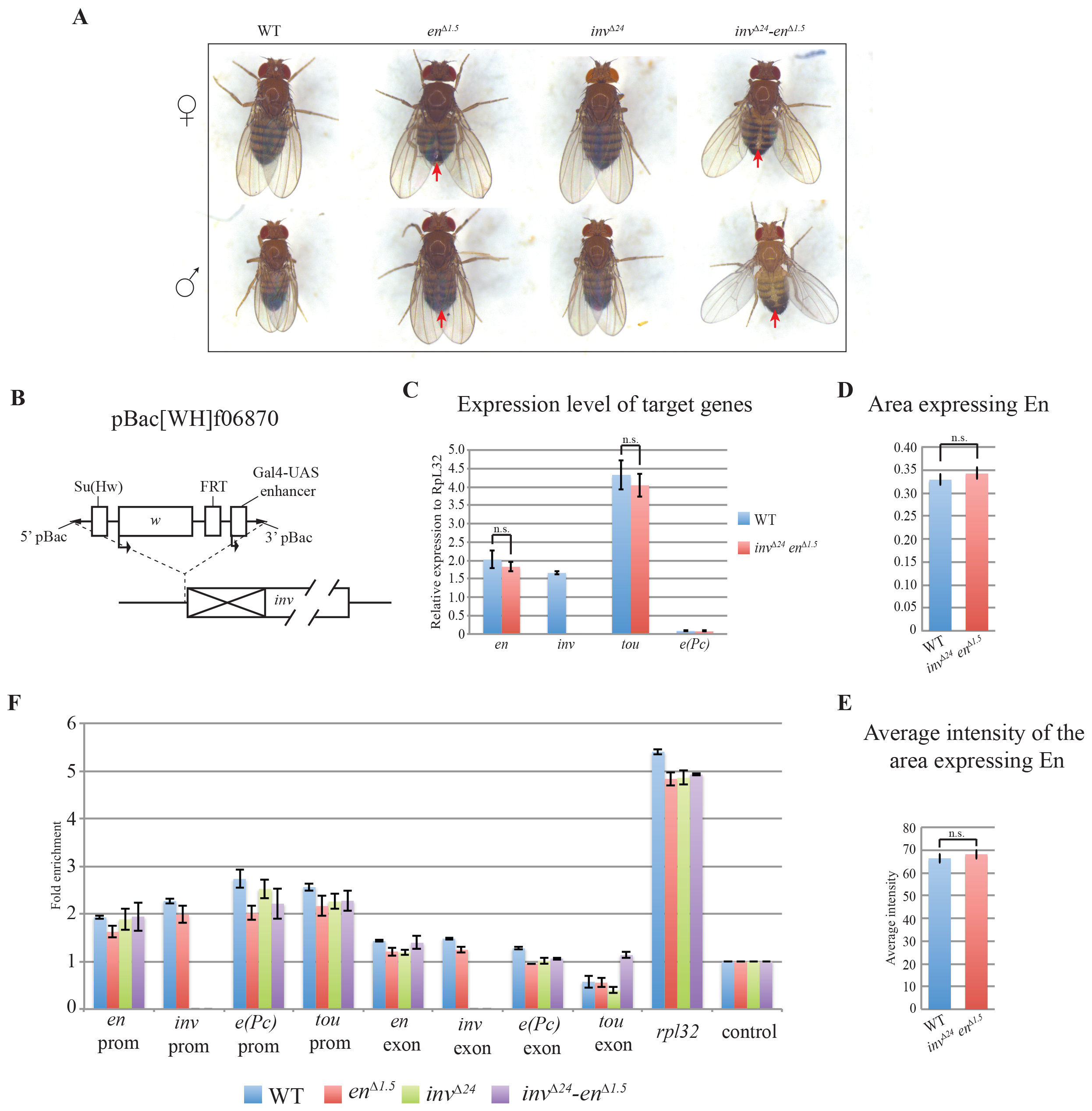

Supplement: S2 Fig — (A) Female (upper panel) and male (lower panel) WT, en∆1.5, inv∆24 and inv∆24 en∆1.5 adults. Defect in fly abdominal cuticle is indicated by red arrows. (B) Schematic of the pBac[WH]f06870 present at the inv∆24 deletion site. inv∆24 was made by recombination between two P[WH] elements, leaving an intact element at the site of the deletion [32]. (C) Quantification of total transcripts of E(Pc), inv, en and tou in WT and inv∆24en∆1.5 relative to total RpL32 transcript in larval wing imaginal disks; data presented is from the average of two independent biological samples with three replicates each (mean±SEM). (D) Quantification of the En expressing area over the total wing disc area, data was collected from 20 imaginal discs of WT and inv∆24 en∆1.5 (mean±SEM). n.s. not significant by student T test. (E) Quantification of fluorescent pixel intensity in the En expressing region in the Wing disc, in the indicated genotypes, data was collected from 15 imaginal discs of WT and inv∆24 en∆1.5 (mean±SEM). (F) Total PolII accumulation quantified on promoters and body of E(Pc), inv, en and tou. The rpl32 gene is used as a positive control. Results are shown as fold enrichment over background signal and are the average of two independent biological samples with three replicates each (mean±SEM). (TIF) [file pgen.1006200.s002.tif]

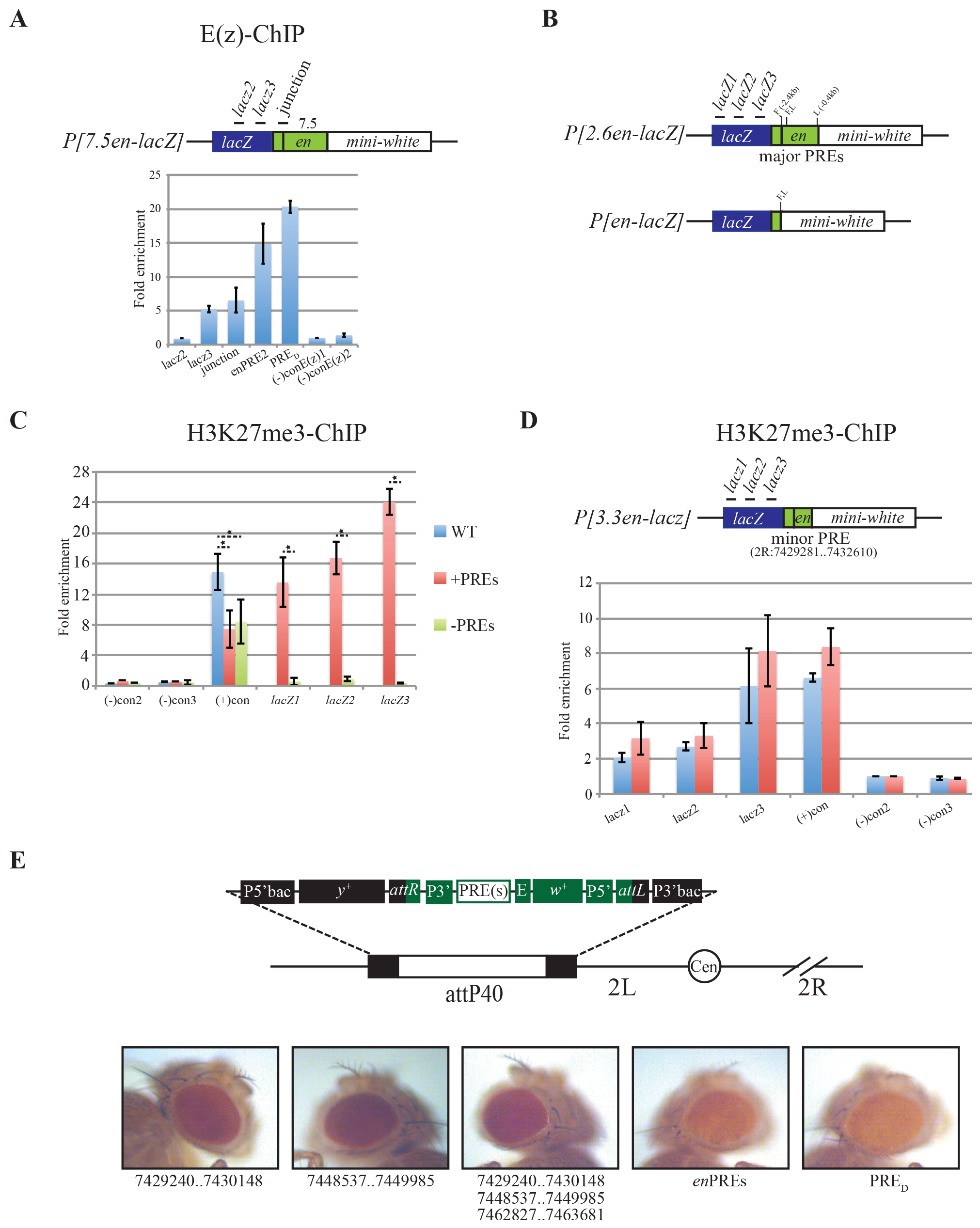

Supplement: S3 Fig — (A) Quantification of E(z) on P[7.5en-lacZ] at the en Promoter-7.5 junction and at the 5’ end of lacZ gene. enPRE2 and PRED (a Ubx PRE) were used as positive control for this experiment. Results are shown as fold enrichment over control signal and are the average of two independent biological samples with three replicates each (mean±SEM). (B) Diagram of the P-element based transgenic construct with and without en characterized PREs [37]. (C) Quantification of H3K27me3 over lacZ, gene. Regions amplified by qPCR are indicated over lacZ gene in the diagram; fragments from e(Pc) and tou were used as negative controls. Results are shown as fold enrichment over background signal and are the average of two independent biological samples with three replicates each (mean±SEM). Statistical analysis of differential H3K27me3 accumulation was performed using Student’s t-test, P-values ≤ 0.05. Only the significant differences with WT are shown with ‘*’. (D) Schematic of a transgene containing a fragment associated with a single weak peak (2R:7429281..7432610, fragment K from Cheng et al., 2014 [32]) is shown in upper panel; quantification of H3K27me3 over lacZ in two transgenic lines containing above transgene is shown. Results are shown as fold enrichment over control signal and are the average of two independent biological samples with three replicates each (mean±SEM). (E) Schematic of the attP40 site after transgene insertion is shown in the top panel, inserted vector is shown in green color. Pictures of the fly eye containing either the strong and weak PcG peaks are shown in the bottom panel. Fragment coordinates used in this assay are also shown. Construct ‘attB-P[acman]-ApR’ [69] was modified by the addition of an eye enhancer from the white gene [32]. Note also that the body color of the enPREs and PRED lines is yellow whereas the other lines have a darker body color. This lighter body color is the result of repression of the y+ transgene present at the attP40 site. (TIF) [file pgen.1006200.s003.tif]

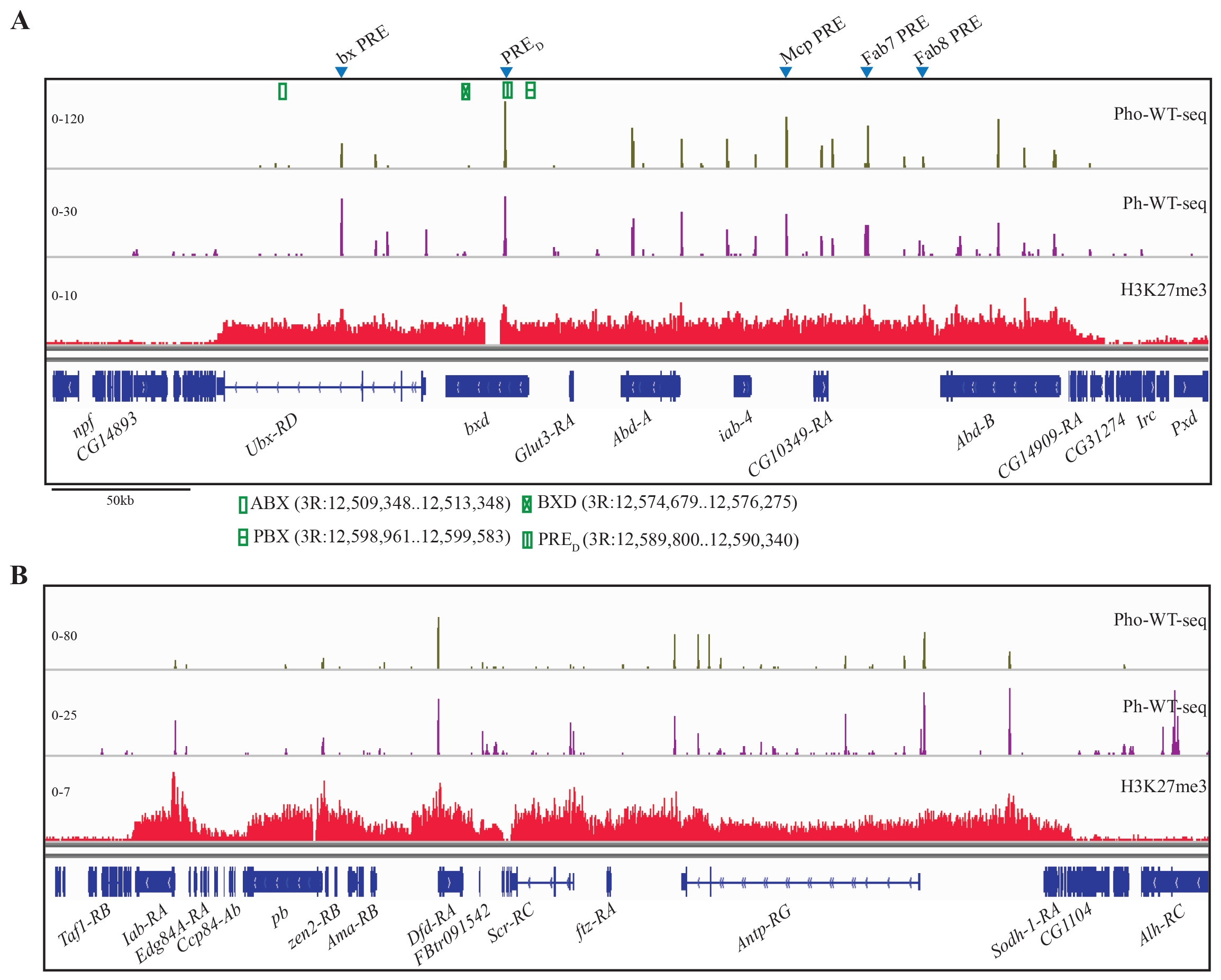

Supplement: S4 Fig — (A) ChIP-seq profiles of Pho, Ph (top two rows) and of H3K27me3 (bottom row) in WT over the Bithorax complex. Well-characterized PREs are indicated with blue arrowheads. Positions of the genes (navy blue) are shown at the very bottom. Regulatory fragments (with corresponding coordinates) identified by Müller and Bienz (1991) [16] are shown in the figure. (B) Profiles of the same over Antp domain. (TIF) [file pgen.1006200.s004.tif]
